# Comparative Risks of Fracture Among Direct Oral Anticoagulants and Warfarin: A Systematic Review and Network Meta-Analysis

**DOI:** 10.3389/fcvm.2022.896952

**Published:** 2022-05-23

**Authors:** Sung Huang Laurent Tsai, Ching-Wei Hu, Shih-Chieh Shao, Eric H. Tischler, Olufunmilayo H. Obisesan, Dominique Vervoort, Wei Cheng Chen, Jiun-Ruey Hu, Liang-Tseng Kuo

**Affiliations:** ^1^Department of Orthopaedic Surgery, Keelung Chang Gung Memorial Hospital, Keelung City, Taiwan; ^2^School of Medicine, Chang Gung University, Taoyuan, Taiwan; ^3^Department of Pharmacy, Keelung Chang Gung Memorial Hospital, Keelung City, Taiwan; ^4^Department of Orthopaedic Surgery and Rehabilitation Medicine, Downstate Medical Center, State University of New York, New York, NY, United States; ^5^Johns Hopkins Bloomberg School of Public Health, Baltimore, MD, United States; ^6^Johns Hopkins School of Medicine, Baltimore, MD, United States; ^7^Department of Orthopaedic Surgery, Chang Gung Memorial Hospital, Taoyuan, Taiwan; ^8^Department of Medicine, Vanderbilt University Medical Center, Nashville, TN, United States; ^9^Division of Sports Medicine, Department of Orthopaedic Surgery, Chang Gung Memorial Hospital, Chiayi, Taiwan

**Keywords:** non-vitamin K antagonist oral anticoagulants, fracture, network meta-analysis, direct-acting oral anticoagulant (DOAC), warfarin, osteoporosis, atrial fibrillation, venous thromboembolism

## Abstract

**Importance:**

Previous studies have shown the effectiveness and safety of direct oral anticoagulants (DOACs), including lower fracture risks, compared to warfarin. However, direct or indirect comparisons between different DOACs are scarce in the literature.

**Objective:**

This study aims to compare fracture risks among different DOACs and warfarin, including apixaban, rivaroxaban, dabigatran, and edoxaban, in patients with non-valvular atrial fibrillation (NVAF) or venous thromboembolism (VTE).

**Methods:**

We searched PubMed/MEDLINE, Embase, Cochrane CENTRAL, and Web of Science for randomized controlled trials and cohort studies comparing the fracture risks among patients who used warfarin or DOACs, up to March 2021. Two authors extracted data and appraised the risk of bias of included studies. The primary outcome was fracture risk. We performed pairwise meta-analyses to compare differences between medications and network meta-analyses using frequentist random-effects models to compare through indirect evidence. We used surface under the cumulative ranking curve (SUCRA) and mean ranks to determine the probability of a DOAC ranking best in terms of fracture risk.

**Results:**

Thirty-one studies were included in the final analysis. Twenty-four randomized controlled trials and seven cohort studies with 455,343 patients were included in the systematic review and network meta-analysis. Compared to warfarin, the risk of any fractures was lowest with apixaban [relative risk (RR) = 0.59; 95% confidence interval (CI): 0.48–0.73], followed by rivaroxaban (RR: 0.72; 95% CI: 0.60–0.86), edoxaban (RR: 0.88; 95% CI: 0.62–1.23), and dabigatran (RR = 0.90; 95% CI: 0.75–1.07). No substantial inconsistency between direct and indirect evidence was detected for all outcomes.

**Conclusions:**

All DOACs were safer than warfarin concerning the risk of fracture; however, apixaban had the lowest relative risk of fracture within the class of DOACs. Further head-to-head prospective studies should confirm the comparative safety profiles of DOACs regarding fractures.

## Key Points

**Question:** What is the comparative risk of fractures in patients using different direct oral anticoagulants (DOACs) and warfarin?
**Findings:** This systematic review with network meta-analysis including 31 studies with 463,495 patients found that, compared to warfarin, the risk of fracture was lowest with apixaban, followed by rivaroxaban, edoxaban, and dabigatran. Our results suggested that among DOACs, apixaban carried the lowest fracture risk.
**Meaning:** DOACs were safer than warfarin with regard to the risk of fracture. Among the DOACs, apixaban had the lowest relative risk of fracture. Healthcare professionals should be informed about different fracture risk profiles associated with different DOACs in order to select the most appropriate DOACs for patients.

## Introduction

As society ages, the prevalence of musculoskeletal and cardiovascular comorbidities increases. Osteoporosis, increasing with age (1), can increase the risk of osteoporotic fracture and subsequent death and disability in the older population (2). The incidence of non-valvular atrial fibrillation (NVAF), another concern in the elderly, continues to increase globally (3). Oral anticoagulants, including vitamin K antagonists (VKAs) and direct oral anticoagulants (DOACs), are recommended for patients with NVAF for the treatment or prevention of stroke and thromboembolism (4).

Warfarin, a classic VKA, has been the mainstay treatment for stroke prevention in patients with AF for decades. Of note, VKA use has been associated with an increase in osteoporotic fragility fractures (5–9). Great concern has been raised by a Medicare population-based study (5), in which AF patients using warfarin for longer than one year show an elevated risk of fragility fracture, compared to those not using warfarin. Bone quality is compromised due to the inhibition of vitamin K-dependent carboxylation of bone metabolism-associated proteins such as osteopontin and matrix Gla (10–15). Despite the potential risk of fragility fracture, warfarin has remained necessary for decades due to the lack of alternatives.

DOACs, recently approved for stroke prevention in AF patients, have been introduced for use as an alternative to warfarin. Given at least equal efficacy in stroke prevention and additional advantages including lower bleeding risk and reduced monitoring requirement compared to warfarin (4), the guidelines of the American College of Cardiology/American Heart Association and the Heart Rhythm Society currently recommend DOACs over warfarin for stroke prevention in NVAF patients (16–18). Consequently, in the United States, DOACs are now more common than VKAs in cardiovascular management (4). Furthermore, DOACs have not been reported to affect bone metabolism proteins (19). Binding et al. report that among 37,350 patients receiving DOACs for over 180 days with no previous use of osteoporotic medications, DOACs are associated with a significantly lower risk of any major osteoporotic fractures, compared to VKAs (6).

As DOACs continue to be a commonplace medication among elderly patients, it is essential to assess the comparative safety profiles, most notably with regard to fractures, within this drug class. Although recent studies have compared fracture risks among the OACs (20–22), the optimal choice of DOAC remains uncertain. Therefore, we performed this systematic review and network meta-analysis to evaluate the network, direct and indirect effects of fracture risk among different DOAC users.

## Methods

### Research Protocol and Search Question

The PICO search protocol framework was followed to address the hypothesis: DOAC use in patients with NVAF or VTE (Population of interest), can lead to a varying reduction in the risk of fractures, depending on which individual DOAC medication is used (Comparator/Intervention). Preferred Reporting Items for Systematic Reviews and Meta-Analyses (PRISMA) statement guidelines were followed for study protocol review and the study was registered in PROSPERO (CRD42020206788).

### Eligibility Criteria and Primary Outcome

Studies were eligible if they met the following criteria: (1) They included adult patients using DOACs for NVAF or VTE. (2) They were observational studies or randomized controlled trials (RCTs). (3) They compared the fracture risk between DOACs and warfarin or other DOACs. Relevant exclusion criteria included: (1) single-arm studies, case reports, small case series of <10 patients, reviews, basic science experiments and animal- or cadaver studies; (2) studies including patients with severe infection or under immunosuppression; and (3) conference abstracts without corresponding full-length papers.

### Search Strategy and Study Selection

On March 27th, 2021, we systematically searched PubMed/MEDLINE, Embase, Ovid, Cochrane Central Register of Controlled Trials (CENTRAL), Web of Science and Scopus for articles using the combination of keywords and medical subject heading (MeSH), adjusted for each database, including: “atrial fibrillation,” “anticoagulant,” “direct oral anticoagulant,” “non-vitamin K antagonist oral anticoagulants,” “vitamin K antagonist oral anticoagulants,” “ warfarin,” “Dabigatran,” “Pradaxa,” “Rivaroxaban,” “Xarelto,” “Apixaban,” “Eliquis,” “Edoxaban,” “Savaysa,” “non-vitamin K antagonist oral anticoagulants,” “novel oral anticoagulants,” “new oral anticoagulants,” “factor Xa inhibitors,” “factor IIa inhibitors,” “fracture,” “osteoporosis” and “osteoporotic fractures.” We also searched the reference lists of the included studies to identify additional studies, and the trial register (clinicaltrials.gov) for any ongoing trials. In addition, we contacted specialists in the field for any ongoing trials or unpublished data. We applied no language restrictions. The detailed search strategy is presented in the Supplementary Table 1.

Two reviewers (SHLT, CWH) independently evaluated eligible studies by their titles and abstracts and then reviewed the full text of relevant articles for further qualification. All disagreements between reviewers were resolved by reaching a consensus through discussion, and a third reviewer (LTK) was consulted where necessary.

### Data Collection and Quality Assessment

Two independent reviewers (SHLT, CWH) extracted all data onto a pre-planned Microsoft Excel spreadsheet (version 16.32). Data fields included study characteristics (authors, year of publication, region of study, data source, study design, period of study), study arms, sample size of overall study and, by study arms, patient age, outcome as defined above, inclusion criteria of each study, specific definition of treatment arm, and source of funding.

The quality of included studies was assessed by two independent reviewers (SHLT, CWH). We evaluated all included RCTs via the RoB (Cochrane risk-of-bias tool for randomized trials) (23), and the non-RCTs via the Newcastle-Ottawa Scale (24). Grade assessment was also performed (25). All discrepancies were resolved by discussion, and a third reviewer (LTK) was consulted where necessary.

### Statistical Analysis and Quantitative Data Synthesis

All statistical analyses were undertaken using Network commands for statistical software package Stata (Version 15). A pairwise function was first used to transform raw data to a contrast-based format and generate treatment effect and standard error for each pairwise comparison. A network meta-analysis was then performed to estimate network meta-analysis models with a frequentist approach derived from graph theoretical methods. The random-effects model was incorporated by adding the estimated heterogeneity τ2, based on the Dersimonian-Laird estimator (26). Subsequently, we examined the structure of our network comparison by applying the netgraph function, with vertices demonstrating treatments and the thickness of edges corresponding to the number of studies.

As a conservative assumption, a random-effects pooled relative risk (RR) with a 95% confidence interval (CI) was calculated to summarize the efficacy of each treatment. Forest plots were constructed to display findings with VKAs as the reference group. Given the I^2^ value increased with the larger populations included in the meta-analysis, τ^2^ was used to measure heterogeneity; 0.04, 0.16, and 0.36 corresponded to a low, moderate, and high degree of heterogeneity, respectively. Subgroup analysis based on treatment comparison was conducted to evaluate heterogeneity within studies. Sensitivity analysis was also performed in the presence of publication bias or significant heterogeneity. We also estimated the probabilities of each treatment being at each rank for each outcome. We obtained a treatment hierarchy using the surface under the cumulative ranking curve (SUCRA) and mean ranks; the SUCRA value is 0 when a treatment is the worst option and 1 when a treatment is the best option (27).

Furthermore, we assessed the potential inconsistency between direct and indirect comparisons using the design-by-treatment interaction model (28), and side-splitting models (29). The design-by-treatment interaction model provides a global assessment of consistency across the entire network. The side-splitting method separates evidence into direct and indirect evidence and then evaluates differences between them (28, 29). We used the Egger's test and a funnel plot to assess small-study bias (30, 31). Symmetry around the effect estimates line indicated lower chance of publication bias or small study effects (32).

### Subgroup Analyses

Where data were available, we planned to perform subgroup analyses including:

1. Fracture location: spinal fracture, hip fracture, and all fractures.
2. DOAC indications: NVAF or VTE/PE.
3. Type of study design: RCTs vs. Non-RCTs.
4. Studies with a given drug dose.
5. Studies with male predominance.
6. Studies with patients aged <65.

## Results

### Literature Search and Selection Process

**Figure 1:**
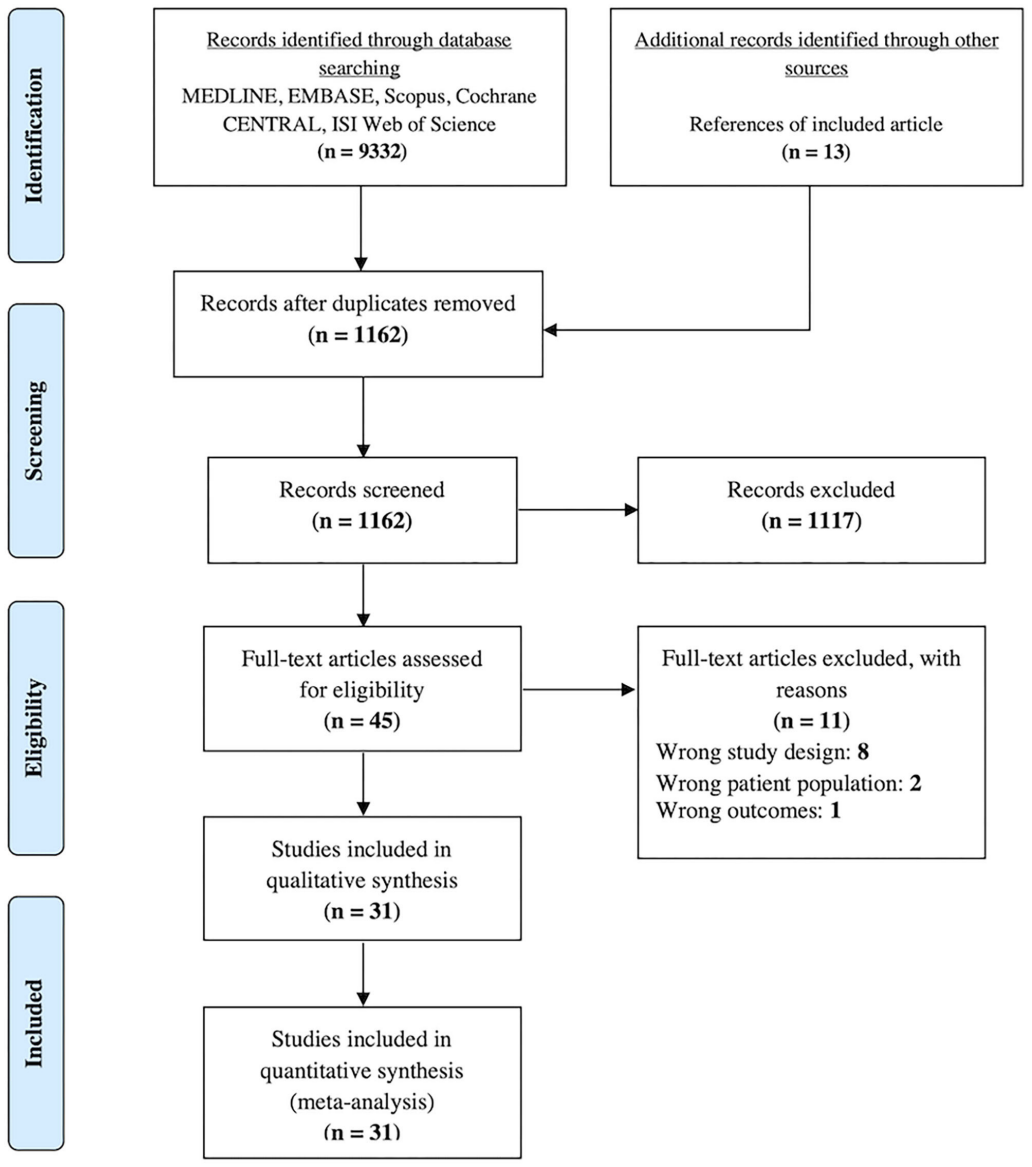
PRISMA flow diagram of the study.

A total of 9,332 articles were identified through the database search. After the removal of duplicates, 1,149 articles remained. An additional 13 articles were identified after checking the reference lists of eligible studies. One thousand one hundred and seventeen articles were excluded by checking the titles and abstracts. After checking the full-text of the remaining 45 articles against the inclusion and exclusion criteria, 11 articles were excluded, whereby eight had the wrong study design, two had the wrong patient population, and one had the wrong outcomes (Supplementary Table 2). Ultimately, 31 studies were included in the network meta-analysis (Figure 1).

### Study Characteristics, Cohort Description and Treatment Definition

**Figure 2:**
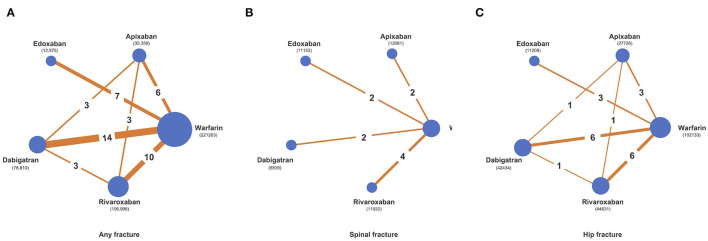
Network diagrams of comparisons of different treatment outcomes in patients receiving warfarin and different direct oral anticoagulants (DOACs). **(A)** Comparisons of all fracture risks in patients receiving warfarin and DOACs. **(B)** Comparisons of spinal fracture risks in patients receiving warfarin and DOACs. **(C)** Comparisons of hip fracture risks in patients receiving warfarin and DOACs.

Our network meta-analysis included 24 randomized controlled trials and seven cohort studies with a total of 455,343 patients receiving five different anticoagulants. Two hundred twenty-one thousand two hundred three patients used warfarin, 78,810 used dabigatran, 106,996 used rivaroxaban, 35,359 used apixaban and the remaining 12,975 patients were edoxaban users. The network graphs are presented in Figures 2A–C, and the main characteristics of the included studies are reported in Table 1. The included studies were conducted in Asia (six studies; 50,203 patients), the Americas (three studies; 270,202 patients), Europe (one study; 14,376 patients), and multinational settings (21 studies; 120,562). The included patients had a median age of 69.05 years (range: 54 to 89 years). A smaller proportion of participants were female (median: 38%) (Table 1). AF and VTE prophylaxis were indications for DOAC use among 93.43% (*N* = 214,198) and 6.57% (*N* = 15,058) of patients, respectively, across the 31 studies. The assumption of transitivity was accepted because no variability was identified in the study and population baselines (Supplementary Tables 3, 4). Supplementary Figure 1 summarizes the detailed risk of bias assessments.

**Table 1:** Characteristics of included studies.

**References**	**Country**	**Study type, LOE**	**Funding**	**Diagnosis**	**Treatment**	**Dosage or INR/Frequency**	**Patient number**	**Fractures** **N (%)**	**Female (%)**	**Age ** **mean ± SD, ** **or range)**
Ezekowitz et al. (33)	Multinational	RCT, I	*Y	≥18 y/o with NVAF	Warfarin	2–3/QD	70	1 (1.43)	15.7	69 ± 8.3
					Dabigatran	50 mg/BID	105	0 (0)	20	70 ± 8.8
					Dabigatran	150 mg/BID	166	0 (0)	18.7	70 ± 8.1
					Dabigatran	300 mg/BID	161	0 (0)	17.4	69.5 ± 8.4
Connolly et al. (34)	Multinational	RCT, I	*Y	≥18 y/o with NVAF, or risk of stroke	Warfarin	2–3/QD	6022	34 (0.56)	36.74	71.6 ± 8.6
					Dabigatran	150 mg/BID	6076	87 (0.71)	36.8	71.5 ± 8.8
					Dabigatran	110 mg/BID	6015	44 (0.73)	35.93	71.4 ± 8.6
Schulman et al. (35)	Multinational	RCT, I	*Y	≥18 y/o with VTE	Warfarin	2–3/QD	1266	2 (0.32)	41.1	54.4 ± 16.2
					Dabigatran	150 mg/BID	1273	4 (0.16)	42	55.0 ± 15.8
U.S. National Library of Medicine (36)	Japan	RCT, I	*Y	≥20 y/o with NVAF	Warfarin	2–3/QD	62	1 (1.61)	8.1	67.4 ± 8.8
					Dabigatran	110 mg/BID	46	0 (0)	21.7	69.9 ± 7.5
					Dabigatran	150 mg/BID	58	0 (0)	8.6	68.3 ± 9.1
Weitz et al. (37)	Multinational	RCT, I	*Y	18–85 y/o with NVAF	Warfarin	NA/QD	250	1 (0.40)	39.6	66.0 ± 8.5
					Edoxaban	30 mg/QD	235	0 (0)	40.4	65.2 ± 8.3
					Edoxaban	30 mg/BID	244	0 (0)	38.5	64.8 ± 8.8
					Edoxaban	60 mg/QD	234	0 (0)	33.8	64.9 ± 8.8
					Edoxaban	60 mg/BID	180	0 (0)	36.7	64.7 ± 9.0
EINSTEIN Investigators et al. (38)	Multinational	RCT, I	*Y	≥18 y/o with VTE	Warfarin + enoxaparin	Warfarin/2–3/QD enoxaparin: Subcutaneous/1 mg/kg/BID	1711	8 (0.47)	43.7	56.4 ± 16.3
					Rivaroxaban	15 mg/BID (for 3 weeks, then 20 mg QD)	1731	6 (0.35)	42.6	55.8 ± 16.4
Chung et al. (39)	Hong Kong South Korea Singapore Taiwan	RCT, I	*Y	18–80 y/o with NVAF	Warfarin	2–3/QD	75	0 (0)	37.3	64.5 ± 9.5
					Edoxaban	30 mg/QD	79	0 (0)	35.4	64.9 ± 9.1
					Edoxaban	60 mg/QD	80	1 (1.25)	31.2	65.9 ± 7.7
Granger et al. (40)	Multinational	RCT, I	*Y	≥18 y/o with NVAF, or risk of stroke	Warfarin	2–3/QD	9052	148 (1.63)	35.0	^∧^70
					Apixaban	2.5 mg or 5 mg/BID	9088	119 (1.30)	35.5	^∧^70
Patel et al. (41)	Multinational	RCT, I	*Y	≥18 y/o with NVAF, or risk of stroke	Warfarin	2–3/QD	7133	116 (1.63)	39.7	^∧^73
					Rivaroxaban	15 mg or 20 mg/BID	7131	82 (1.15)	39.7	^∧^73
EINSTEIN-PE Investigators et al. (42)	Multinational	RCT, I	*Y	≥18 y/o with PE	Warfarin + enoxaparin	Warfarin/2–3/QD enoxaparin: Subcutaneous/1 mg/kg/BID	2413	9 (0.37)	48.3	57.5 ± 7.2
					Rivaroxaban	15 mg/BID (for 3 weeks, then 20 mg QD)	2419	15 (0.62)	45.9	57.9 ± 7.3
Hori et al. (43)	Japan	RCT, I	*Y	≥20 y/o with NVAF, or risk of stroke	Warfarin	2–3/QD	639	10 (1.56)	21.8	71.2 (43–90)
					Rivaroxaban	15 mg/BID	639	10 (1.56)	17.1	71.0 (34–89)
Hokusai-VTE Investigators et al. (44)	Multinational	RCT, I	*Y	≥18 y/o with PE or DVT	Warfarin	2–3/QD	4122	48 (1.16)	42.8	55.9 ± 16.2
					Edoxaban	60 mg/QD	4118	45 (1.09)	42.7	55.7 ± 16.3
Agnelli et al. (45)	Multinational	RCT, I	*Y	≥18 y/o with PE or DVT	Warfarin + enoxaparin	Warfarin/2–3/QD enoxaparin: Subcutaneous/1 mg/kg/BID	2704	13 (0.48)	40.9	56.7 ± 16.0
					Apixaban	10 mg/BID (for 1 week, then 5 mg BID)	2691	6 (0.22)	41.7	57.2 ± 16.0
Giugliano et al. (46)	Multinational	RCT, I	*Y	≥21 y/o with NVAF or risk of stroke	Warfarin	2–3/QD	7036	240 (3.41)	37.5	^∧^72
					Edoxaban	30 mg/QD	7034	223 (3.17)	38.8	^∧^72
					Edoxaban	60 mg/QD	7035	429 (2.93)	37.9	^∧^72
Schulman et al. (47)	Multinational	RCT, I	*Y	≥18 y/o with PE or DVT	Warfarin	2–3/QD	1426	12 (0.84)	38.9	53.9 ± 15.3
					Dabigatran	150 mg/BID	1430	6 (0.42)	39.1	55.4 ± 15.0
Schulman et al. (48)	Multinational	RCT, I	*Y	≥18 y/o with PE or DVT	Warfarin	2–3/QD	1288	3 (0.23)	39.8	55.1 ± 16.3
					Dabigatran	150 mg/BID	1280	3 (0.23)	39	54.7 ± 16.2
Gibson et al. (49)	Multinational	RCT, I	*Y	≥18 y/o with NVAF and PCI	Warfarin + aspirin + clopidogrel	2–3/QD 75–100 mg/QD 75 mg/QD	706	6 (0.85)	26.6	69.9 ± 8.7
					Rivaroxaban + aspirin + clopidogrel	2.5 mg /BID 75–100 mg/QD 75 mg/QD	709	2 (0.28)	24.5	70.0 ± 9.1
					Rivaroxaban + clopidogrel	15 mg /QD 75 mg/QD	709	6 (0.85)	25.5	70.4 ± 9.1
Goette et al. (50)	Multinational	RCT, I	*Y	≥18 y/o with NVAF	Warfarin	2–3/QD	1104	0 (0)	35	64.2 ± 10.8
					Edoxaban	60 mg/QD	1095	1 (0.09)	34	64.3 ± 10.3
Piazza et al. (51)	Multinational	RCT, I	*Y	≥18 y/o with DVT	Warfarin	2–3/QD	28	2 (7.14)	25	53.1 ± 12
					Edoxapan	90 mg/QD (for 10 days, then 60 mg QD for 90 days)	56	0 (0)	26.8	55.6 ± 14.1
Bengtson et al. (52)	USA	CS, IIa	Y	Stroke prevention for non-AF	Warfarin	NA	37707	275 (0.73)	38.8	70.8 ± 12.1
					Dabigatran	75 mg or ^+^150 mg/NA	18981	108 (0.57)	36.2	68.5 ± 12.3
Calkins et al. (53)	Multinational	RCT, I	*Y	≥18 y/o with NVAF	Warfarin	2–3/QD	318	0 (0)	23	59.3 ± 10.3
					Dabigatran	150 mg/BID	317	0.32	27.4	59.1 ± 10.4
Cannon et al. (54)	Multinational	RCT, I	*Y	≥18 y/o with NVAF and PCI (within previous 120 h)	Warfarin + aspirin + clopidogrel	2–3/QD ≤ 100 mg/QD 90 mg/BID	981	13 (1.33)	23.5	71.7 ± 8.9
					Dabigatran + clopidogrel or ticagrelor	110 mg/BID 75 mg/QD 90 mg/BID	981	9 (0.92)	25.8	71.5 ± 8.9
					Dabigatran + clopidogrel or ticagrelor	150 mg/BID 75 mg/QD 90 mg/BID	763	6 (0.79)	22.4	68.6 ± 7.7
Lucenteforte et al. (55)	Italy	CS, IIa	Y	Patients with OACs	Warfarin	NA	13091	153 (1.17)	48.29	NA
					DOAC (D, R, A)	NA	3759	41 (1.09)	51.08	
					Direct Xa inhibitor (R,A)	NA	2474	26 (1.05)	51.70	
					Dabigatran	NA	1285	15 (1.16)	49.88	
Norby et al. (56)	USA	CS, IIa	Y	22–99 y/o with NVAF	Warfarin	NA	45496	408 (0.90)	40.1	71.1 ± 12.5
					Rivaroxaban	10 or 15 mg or 20 mg/NA	32495	194 (0.60)	38.7	69.3 ± 12.2
Ezekowitz et al. (57)	Multinational	RCT, I	*Y	≥18 y/o with NVAF within 48 h	Warfarin	2–3/QD	747	0 (0)	33.5	64.5 ± 12.8
					Apixaban	5 mg/BID	753	3 (0.40)	32.9	64.7 ± 12.2
Hohnloserm et al. (58)	Multinational	RCT, I	*Y	≥18 y/o with NVAF scheduled for first or repeated catheter ablation	Warfarin	2–3/QD	203	0 (0)	26.6	61 (52–67)
					Edoxaban	60 mg/QD	411	1 (0.24)	29.4	60 (53–67)
Ferro et al. (59)	Multinational	RCT, I	*Y	18–78 y/o cerebral venous thrombosis	Warfarin	2–3/QD	60	1 (1.67)	55	45.2 ± 13.8
					Dabigatran	150 mg/BID	60	0 (0)	55	45.2 ± 13.8
Huang et al. (20)	Taiwan	CS, IIa	NA	≥20 y/o with newly NVAF	Warfarin	NA	9707	1009 (10.39)	41.1	71.3 ± 11.5
					DOAC (D, R, A)	NA	9707	737 (7.59)	40.8	72.4 ± 10.7
					Warfarin	NA	5796	660 (11.39)	37.6	73.3 ± 11.2
					Dabigatran	NA	5796	535 (9.23)	36.6	73.6 ± 10.1
					Warfarin	NA	7287	831 (11.40)	42.7	73.2 ± 10.9
					Rivaroxaban	NA	7287	530 (7.27)	42.4	73.9 ± 10.3
					Warfarin	NA	1761	204 (11.58)	42.8	75.1 ± 11.1
					Apixaban	NA	1761	89 (5.05)	42.1	75.0 ± 10.0
Lutsey et al. (60)	USA	CS, IIa	Y	18–99 y/o with NVAF	Warfarin	NA	55826	2829 (5.07)	*W: 38.8 *D: 34.9 *R: 38.1 *A: 39.9	*W:70.2 ± 12.3 *D: 67.0 ± 12.4 *R: 67.7 ± 12.3 *A: 69.1 ± 12.6
					DOACs (D, R, A)	NA	55826	2685 (4.81)		
					Warfarin	NA	31612	1803 (5.70)		
					Dabigatran	75 mg or 150 mg/NA	31612	1764 (5.58)		
					Warfarin	NA	32440	1494 (4.60)		
					Rivaroxaban	10 or 15 mg or 20 mg/NA	32440	1124 (3.46)		
					Warfarin	NA	15645	521 (3.33)		
					Apixaban	2.5 mg or 5 mg/NA	15645	396 (2.53)		
					Dabigatran	75 mg or 150 mg/NA	12572	510 (4.06)		
					Rivaroxaban	10 or 15 mg or 20 mg/NA	12572	543 (4.32)		
					Apixaban	2.5 or 5 mg/NA	16621	401 (2.41)		
					Rivaroxaban	10 or 1 5mg or 20 mg/NA	16621	394 (2.37)		
					Apixaban	2.5 mg or 5 mg/NA	5112	153 (2.99)		
					Dabigatran	75 mg or 150 mg/NA	5112	160 (3.13)		
Wang et al. (61)	China	CS, IIa	NA	≥60 y/o with DM and NVAF	Warfarin	NA	383	13 (3.39)	45	68.69 ± 5.56
					Rivaroxaban	20 mg/QD	201	1 (0.50)	21	69.52 ± 4.55
Lau et al. (21)	Hong Kong	CS, IIa	Y	NVAF	Warfarin	NA	9541	196 (2.05)	45.2	73.1 ± 11.4
					Dabigatran	NA	6867	95 (1.38)	49.2	74.4 ± 10.0
					Rivaroxaban	NA	3866	57 (1.47)	49.5	75.0 ± 10.3
					Apixaban	NA	3241	53 (1.64)	51.8	77.9 ± 10.3

*Treatment group before characteristic match; ^+^: Majority; ^∧^: Median; *Y: funding from pharmaceutical company.

*LOE, Level of evidence according to Halperin et al. (62); CS, cohort study; RCT, randomized controlled trial*.

*NVAF, non-valvular atrial fibrillation; PE, Pulmonary embolism; VTE, venous thromboembolism; PCI, Percutaneous coronary intervention; QD, Once daily; BID, Twice daily; INR, International normalized ratio; NA, not applicable; N, no; OACs, oral anticoagulants; DOAC, direct oral anticoagulant; D, dabigatran; R, rivaroxaban; A, apixaban; E, edoxaban; y/o, year-old*.

### Methodological Quality and Assessment of Risk of Bias

The main sources of RoB in the included RCTs were blinding of participants, personnel, and incomplete outcome data. Connolly et al. (34), EINSTEIN Investigators et al. (38), and EINSTEIN–PE Investigators et al. (42) had a high risk of performance bias, while Gibson et al. (49), Hohnloser et al. (58), Piazza et al. (51) and Weitz et al. (37) had a risk of attrition bias (Supplementary Figures 1A,B). The quality of non-RCTs was fairly good (Supplementary Table 5). Most studies had funding from multinational pharmaceutical companies. Only Huang et al. (63) and Wang et al. (61) did not report external funding.

### Fracture Risk

**Figure 3:**
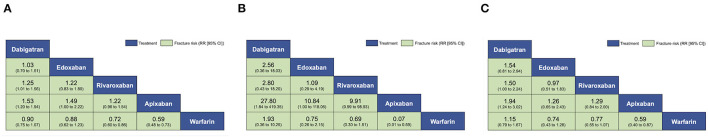
Pooled estimates of the network meta-analysis. Comparisons, column vs. row, should be read from left to right and are ordered relative to overall effectiveness. **(A)** Pooled risk ratios (95% confidence intervals [CI]) for all fractures. **(B)** Pooled risk ratios (95% CI) for spinal fractures. **(C)** Pooled risk ratios (95% CI) for hip fractures.

We summarized our random-effects network meta-analysis and pairwise comparison of fracture risks in Figure 3; Supplementary Table 6. We ranked the risk of any fractures of DOACs against warfarin and the SUCRA probability (Supplementary Figures 3, 4; Supplementary Table 7).

### Any Fracture Risk

This outcome was reported by 31 studies with 455,343 participants. The overall structure is shown in Figure 2A. VKA users had 5,553 fractures (5,553/2,21,203, 2.51%), dabigatran users had 2,578 fractures (2,578/78,810, 3.27%), rivaroxaban users had 2,025 fractures (2,025/1,06,996, 1.89%), apixaban users had 666 fractures (666/35,359, 1.88%) and edoxaban users had 254 fractures (254/12,975, 1.96%). Comparing network estimates of fracture risk between DOACs and warfarin, apixaban users (RR: 0.59; 95% CI: 0.50 to 0.71) and rivaroxaban users (RR: 0.72; 95% CI: 0.64 to 0.84) showed a statistically significant reduction in fracture risk, compared to warfarin users. No significant fracture risk reduction was observed among edoxaban, dabigatran, and warfarin users (Figure 3A). In terms of any fracture risk, apixaban (SUCRA = 98.0%) was most likely to be ranked the best, followed by rivaroxaban (SUCRA = 71.6%) (Supplementary Figures 3A,B, 4A; Supplementary Table 7).

### Spine Fracture Risk

This outcome was reported in 10 studies with 83,842 participants (34, 38, 40–46, 54). The overall structure is shown in Figure 2B. VKA users had 61 fractures (61/4,1849, 0.15%), dabigatran users had 5 fractures (5/6,839, 0.07%), rivaroxaban users had 15 fractures (15/11,920, 0.13%) and edoxaban users had 16 fractures (16/11,153, 0.14%). No spinal fracture event was reported among apixaban users. Pooled estimates revealed no significant differences among apixaban users (RR: 0.07; 95% CI: 0.01 to 0.57), rivaroxaban users (RR: 0.75; 95% CI: 0.34 to 1.69), edoxaban users (RR: 0.78; 95% CI: 0.33 to 1.81) and dabigatran users (RR: 1.72; 95% CI: 0.32 to 9.17), when compared to warfarin users (Figure 3B). Apixaban (SUCRA = 98.5%) was most likely to be ranked the best in terms of risks for spine fracture (Supplementary Figures 3A,B, 4B; Supplementary Table 7).

### Hip Fracture Risk

This outcome was reported in 16 studies with 228,133 participants (34, 35, 38, 40–42, 44–49, 51, 54, 60, 61). The overall structure is shown in Figure 2C. VKA users had 453 fractures (453/1,02,133, 0.44%), dabigatran users had 195 fractures (195/42,434, 0.46%), rivaroxaban users had 158 fractures (158/44,631, 0.35%), apixaban users had 69 fractures (69/27,726, 0.25%), and edoxaban users had 36 fractures (36/11,209, 0.32%). Overall, apixaban users generated the lowest pooled fracture risk estimate (RR: 0.56; 95% CI: 0.43 to 0.74), followed by rivaroxaban users (RR: 0.73; 95% CI: 0.60 to 0.88), edoxaban users (RR: 0.73; 95% CI: 0.47 to 1.12) and dabigatran users (RR: 1.06; 95% CI: 0.89 to 1.26), compared to warfarin users (Figure 3C). Apixaban (SUCRA = 90.4%) was most likely to be ranked the best in terms of risks for hip fracture (Supplementary Figures 3A,B, 4C; Supplementary Table 7).

### Subgroup Analyses

The detailed results of subgroup analyses were presented in Supplementary Tables 8–10. Of note, in 22 studies with the indication of NVAF, apixaban reported the lowest fracture risk compared to warfarin (RR: 0.59; 95% CI: 0.58 to 0.75), followed by rivaroxaban (RR: 0.70; 95% CI: 0.58 to 0.85, Supplementary Table 10). Eight studies reported the indication of VTE, none of the DOACs were statistically significant in fracture reduction compared to warfarin. The results were similar in the subgroup of patients older than 65 and male predominant studies. Advanced age and male sex are both common characteristics of the NVAF population, with both subgroups concluding the lowest fracture risk in apixaban users (Supplementary Table 10).

### Exploration for Inconsistency and Publication Bias

We found no evidence of global inconsistency in any of the outcomes using the design-by-treatment interaction models (Supplementary Table 11A). Furthermore, no substantial inconsistency between direct and indirect comparisons was observed in the side-splitting models (Supplementary Table 11B). Supplementary Figure 4A shows the comparison-adjusted funnel plots of fracture risks in the included studies, which revealed no significant funnel plot asymmetry. Lastly, the Egger test revealed no evidence of small-study bias (Supplementary Figure 4A).

### Grade

We incorporated the GRADE judgments for network estimates of fracture risks. The certainty of evidence for the risk between anticoagulants varied; it was moderate for most of the comparisons involving DOACs against warfarin with regards to risks for any fracture, spine fracture, and hip fracture. The certainty of evidence was mostly moderate to low for the comparisons between different DOACs (Supplementary Tables 12, 13A–C).

## Discussion

The current study aimed to identify fracture risks among patients prescribed DOACs and warfarin. The principal findings of this study were that patients who were prescribed apixaban carried the lowest fracture risk, followed by rivaroxaban, edoxaban and dabigatran, compared to patients prescribed warfarin. When assessing future fracture risk, it is crucial to consider both patient medication and medical history, given that 30% of patients presenting with a proximal femoral fracture receive anticoagulation therapy (64). Owens et al. reported that dabigatran, rivaroxaban, and apixaban might be used safely in NVAF patients with specific valvular heart diseases including aortic stenosis, aortic regurgitation, and mitral regurgitation (65). By contrast, patients with moderate to severe mitral stenosis or mechanical valves should continue to receive warfarin, as these patients have routinely been excluded from NVAF clinical trials (65). Furthermore, NVAF or VTE patients may require long-term anticoagulation therapy. Previous studies have reported that long-term exposure to VKAs is associated with an increased risk of fractures (66). These findings could be an important reference for clinicians when evaluation of fracture risk is necessary for patients at high risk of fractures, such as the elderly, who need to be on anticoagulation for NVAF.

As regards DOAC use and fracture risk, the literature remains conflicted. Both Lau et al. and Lutsey et al. report that DOACs carry a lower risk of fractures in patients with NVAF in the US and Hong-Kong, respectively, compared to warfarin (21, 60). However, Lucenteforte et al. find no differences in fracture risk between DOACs and VKA in patients with NVAF in Italy (55). These discrepancies in results may be attributed to heterogeneity of the study populations and the studies' power to detect event rate differences, whereby new RCTs and cohort studies that have since appeared add to our understanding of DOACs, especially those new to us (apixaban). Our systematic review and network meta-analysis evaluated 24 RCTs and seven cohort studies and observed that DOAC use was associated with a 21% risk reduction in reported fractures, compared to patients receiving warfarin.

Physiologically, the difference in fracture risk between DOACs and VKAs may be attributed to pharmacologic bone mineral density. Extensive literature survey reveals that both hip and vertebral fractures are most common among osteoporotic patients (67). VKAs inhibit the carboxylation of vitamin K-dependent bone mineralization proteins, including osteocalcin, matrix Gla protein, and periostin, increasing fracture risk (10, 11, 14, 68). Inhibition of osteocalcin carboxylation reduces adherence to calcium and hydroxyapatite, decreasing bone mineral density (BMD) and increasing the risk of osteoporosis (69). In animal studies, Fusaro et al. determined that among rats administered warfarin, a significant decrease in histomorphometric bone volume and increase in trabecular separation was observed, compared to both Dabigatran and placebo groups (11). In human studies, Rezaieyazdi et al. observed a marked reduction in BMD (g/cm^2^) and T-score of the lumbar spine among 70 rheumatic valvular heart disease patients taking warfarin, compared to controls (*P* = 0.048) (12). Warfarin use was the only risk factor of significant importance on spinal T-score (*P* < 0.03) (12). These findings support the utility of DOACs in decreasing fractures, compared to VKAs.

Kuo et al. (22) queried the Taiwan National Health Insurance database and reported that among 56,795 patients prescribed DOACs, dabigatran users show a lower incidence of osteoporotic fracture and spine fracture than patients receiving standard-dose rivaroxaban and apixaban. Our findings regarding the lower fracture risk of DOACs compared to warfarin have supported the already favorable clinical efficacy and side effect profiles of DOACs, compared to warfarin. Apixaban is superior to warfarin in the prevention of stroke and systemic embolism. The rates of stroke and ICH are both significantly lower in the ARISTOTLE trial (40). In a meta-analysis of 28 RCTs comparing DOACs with warfarin all DOACs have a higher rate of major GI bleeding, except apixaban (70). Our study findings also showed a statistically significant, lowered risk of fracture for apixaban, compared to warfarin. This lends support to the safety of apixaban use in elderly patients with regard to GI bleeding profiles, especially if these patients are at high risk of fracture.

Although DOACs have been reported to decrease fracture risk with protective bone mineralization properties compared to VKAs, not all fractures pose the same risk; therefore, subgroup analysis of anatomic fracture location is critical. Concerning hip fractures, our study determined that compared to warfarin, all of the DOACs except dabigatran exhibited a decreased hip fracture risk in the following descending order: apixaban, rivaroxaban, and edoxaban. Consistent with our findings, Huang et al. report a statistically significant risk reduction in hip fractures among adult users of DOACs, compared to VKAs, with varying risk reduction rates among the DOACs (63). Further research is required to determine the pharmacological mechanism of apixaban that contributes to fracture risk reduction in comparison to other DOACs.

Unlike osteoporotic hip and spine fractures among the elderly, trauma is typically associated with a high energy mechanism in the younger population with fewer comorbidities that do not require anticoagulation. Our findings showed that when only patients below 65 were included, no significant effect was seen among the DOACs, compared to warfarin. Most current literature focuses on patients with pre-existing comorbidities requiring anticoagulation treatment (71, 72). Second, most NVAF or VTE patients may require long-term anticoagulation therapy, and previous studies have indicated that long-term exposure to VKAs is associated with an increased risk of fractures (69, 73). In our study, we found that long-term DOAC exposure of at least one year also decreased the risk of fractures by 21%, compared to warfarin. Although the fracture types, treatment duration, and patients' sex or age varied among the included studies, the resulting overall robustness was proven by the subgroup analyses. The older female population is already known to be associated with increased fracture risk (74). We found that the female- and male population achieved similar effects when using DOACs to decrease fracture risks. Apixaban had the lowest fracture risks (RR: 0.55; 95% CI: 0.46 to 0.65), compared to warfarin, in the predominantly male studies. When we included only studies with younger patients (aged < 65), no significant effect was seen among the DOACs, compared to warfarin, which may be explained by the diminished overall sample size as a result of including only these studies. Most studies evaluated patients older than 65.

To the best of our knowledge, at present, this is the most comprehensive, up-to-date network meta-analysis to analyze the fracture risk among patients receiving DOACs and VKA. However, some limitations must be addressed. It should be noted that there was cohort heterogeneity among the studies. Although over 90% of studies analyzed involved AF patients, some study cohorts included trauma patients who received oral anticoagulation for thromboprophylaxis. Reassuringly, our sensitivity analysis revealed consistent results in patients with varying indications for anticoagulation. It should also be noted that potential confounders, including age, sex, race, and comorbidities, were adjusted for, using propensity score matching to allow for robust, accurate data comparison. Additionally, most studies did not provide BMD data as it rarely was a primary or secondary outcome; therefore, further research is required to quantify the association of oral anticoagulants with measured changes in T-score. Future meta-analyses on individual-level participant data and head-to-head prospective studies will be beneficial to confirm the findings above.

## Conclusion

In summary, this network meta-analysis demonstrated that apixaban had the lowest pooled fracture risk, compared to other DOACs, and that the four major DOACs had lower fracture risk than warfarin. Similar results were found in sensitivity analyses with lower heterogeneity and inconsistency. These findings might benefit clinical practice for the individualized use of anticoagulants; however, future, large head-to-head prospective studies are required to validate these findings.

## Data Availability Statement

The original contributions presented in the study are included in the article/Supplementary Material, further inquiries can be directed to the corresponding author/s.

## Author Contributions

ST, C-WH, S-CS, and L-TK conceived and designed the study, interpreted data, and contributed to the final version of this report. ST and C-WH selected the articles and extracted the data. ST and L-TK analyzed the data. ST, C-WH, and L-TK wrote the draft. ET, OO, DV, WC, and J-RH made critical revisions. All authors agreed with the results and conclusions reported. All authors contributed to the article and approved the submitted version.

## Conflict of Interest

The authors declare that the research was conducted in the absence of any commercial or financial relationships that could be construed as a potential conflict of interest.

## Publisher's Note

All claims expressed in this article are solely those of the authors and do not necessarily represent those of their affiliated organizations, or those of the publisher, the editors and the reviewers. Any product that may be evaluated in this article, or claim that may be made by its manufacturer, is not guaranteed or endorsed by the publisher.

## Abbreviations

CI: confidence interval
DOAC: direct oral anticoagulants
NVAF: non-valvular atrial fibrillation
PICO: Patient-Intervention-Comparison-Outcome
PRISMA: Preferred Reporting Items for Systematic Reviews and Meta-Analyses
RR: relative risk
VKA: vitamin K antagonists.
