# Supplementary material for: Comparative Risks of Fracture Among Direct Oral Anticoagulants and Warfarin: A Systematic Review and Network Meta-Analysis
Source: Front Cardiovasc Med. 2022 May 23;9:896952. doi: 10.3389/fcvm.2022.896952 (PMC9168033; doi:10.3389/fcvm.2022.896952)
Supplement: Supplementary file 1 [file Data_Sheet_1.docx]

**SUPPLEMENTARY**

**The following material accompanies the article “Comparative Risks of Fracture among Direct Oral Anticoagulants and warfarin: A Systematic Review and Network Meta-analysis”**

**Table of Content**

| **Supplementary Figure 1** Risk of bias assessment of the included studies | P3 |
| --- | --- |
| **Supplementary Figure 2** Rank plot | P5 |
| **Supplementary Figure 3** Forest plot of network meta-analysis results | P7 |
| **Supplementary Figure 4:** Publication bias | P10 |
| **Supplementary Table 1** Electronic database search strategy | P12 |
| **Supplementary Table 2** Excluded studies with reasons | P15 |
| **Supplementary Table 3** Descriptions of the included studies | P16 |
| **Supplementary Table 4** Patient characteristics included in the studies | P21 |
| **Supplementary Table 5** Risk of bias assessment according to Newcastle-Ottawa Quality Assessment Scale for cohort studies | P23 |
| **Supplementary Table 6** Pairwise meta-analysis of risk ratio (95% CI) for fractures | P24 |
| **Supplementary Table 7** Mean rank and SUCRA (surface under the cumulative ranking curve) of five anticoagulants for risk of fractures | P25 |
| **Supplementary Table 8** Studies involved in sensitivity analysis | P26 |
| **Supplementary Table 9** Subgroup Pairwise meta-analysis of risk ratio (95% CI) for any fracture | P27 |
| **Supplementary Table 10** Subgroup Network meta-analysis of risk ratio (95% CI) for any fracture | P28 |
| **Supplementary Table 11** Assessment of inconsistency | P29 |
| **Supplementary Table 12** Assessment of the quality of included studies by GRADE | P31 |
| **Supplementary Table 13** GRADE approach for rating the quality of treatment effect estimate | P32 |

**Supplementary Figure 1** Risk of bias assessment of the included studies

**Supplementary Figure 1A** Summary of the risk of bias items of RCTs


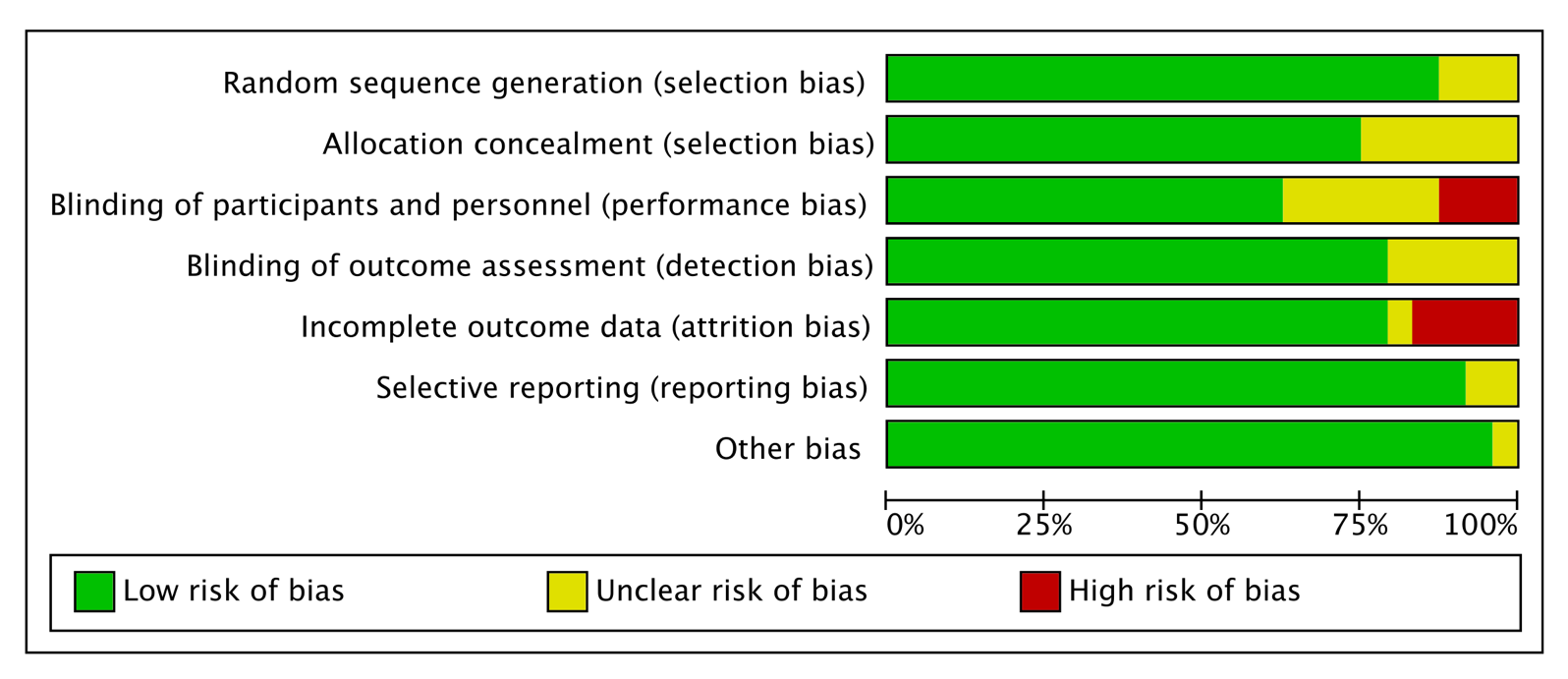


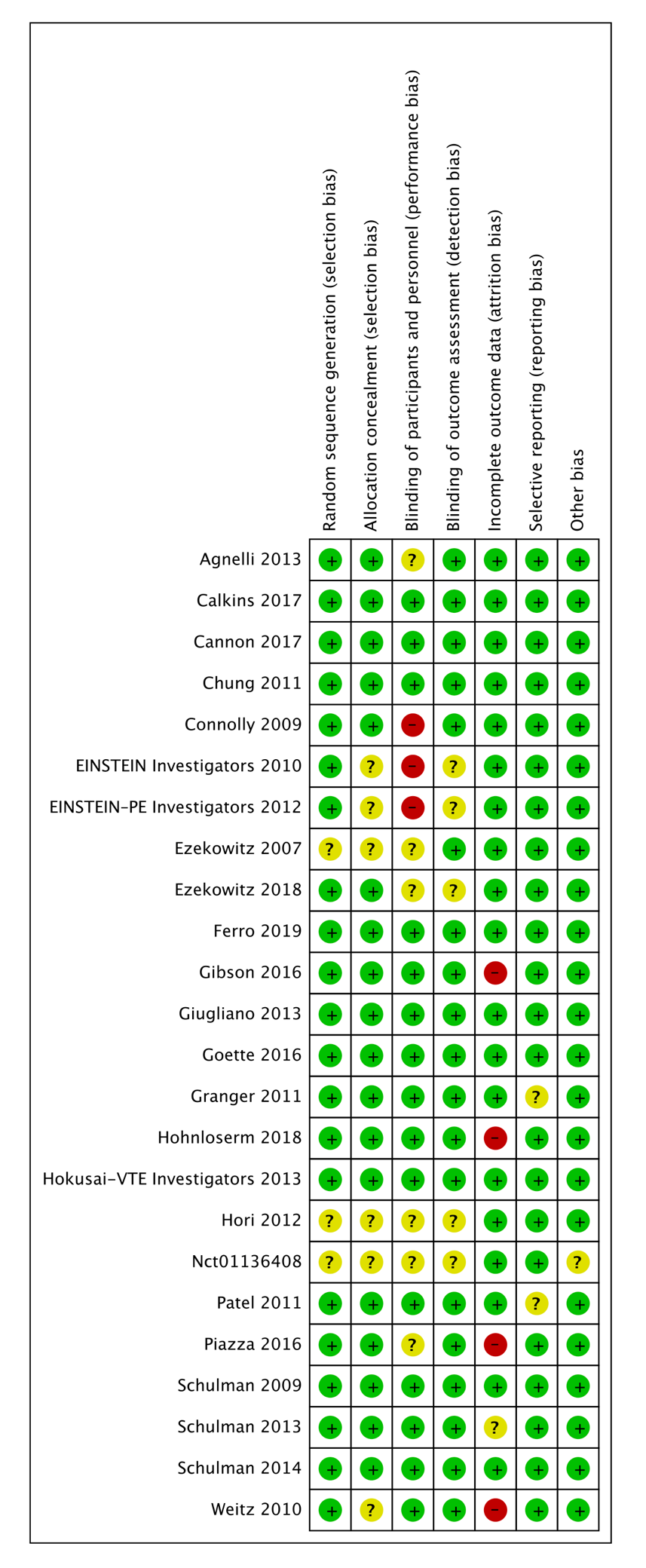
**Supplementary Figure 1B** Detailed risk of bias assessment of the individual RCTs

**Supplementary Figure 2** Rank plot

**Supplementary Figure 2A** Rank plot of five anticoagulants for risk of fractures

| **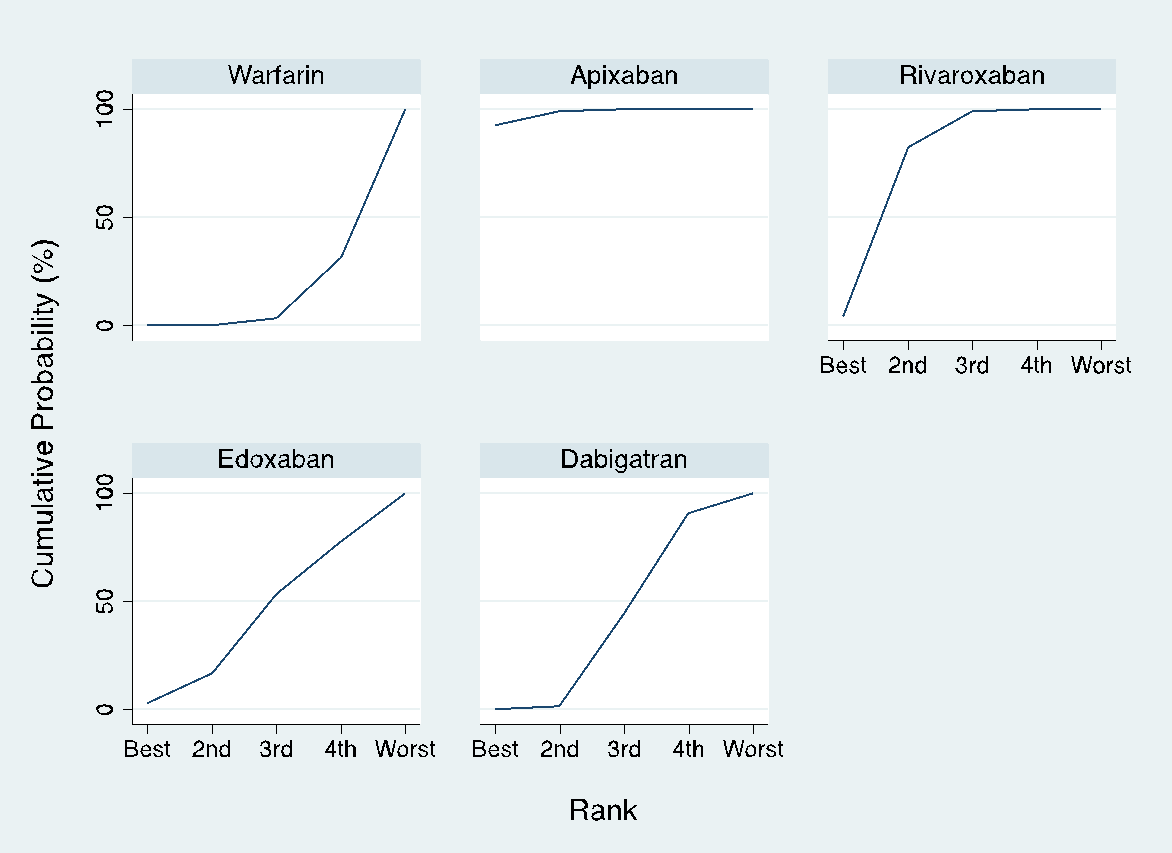** | **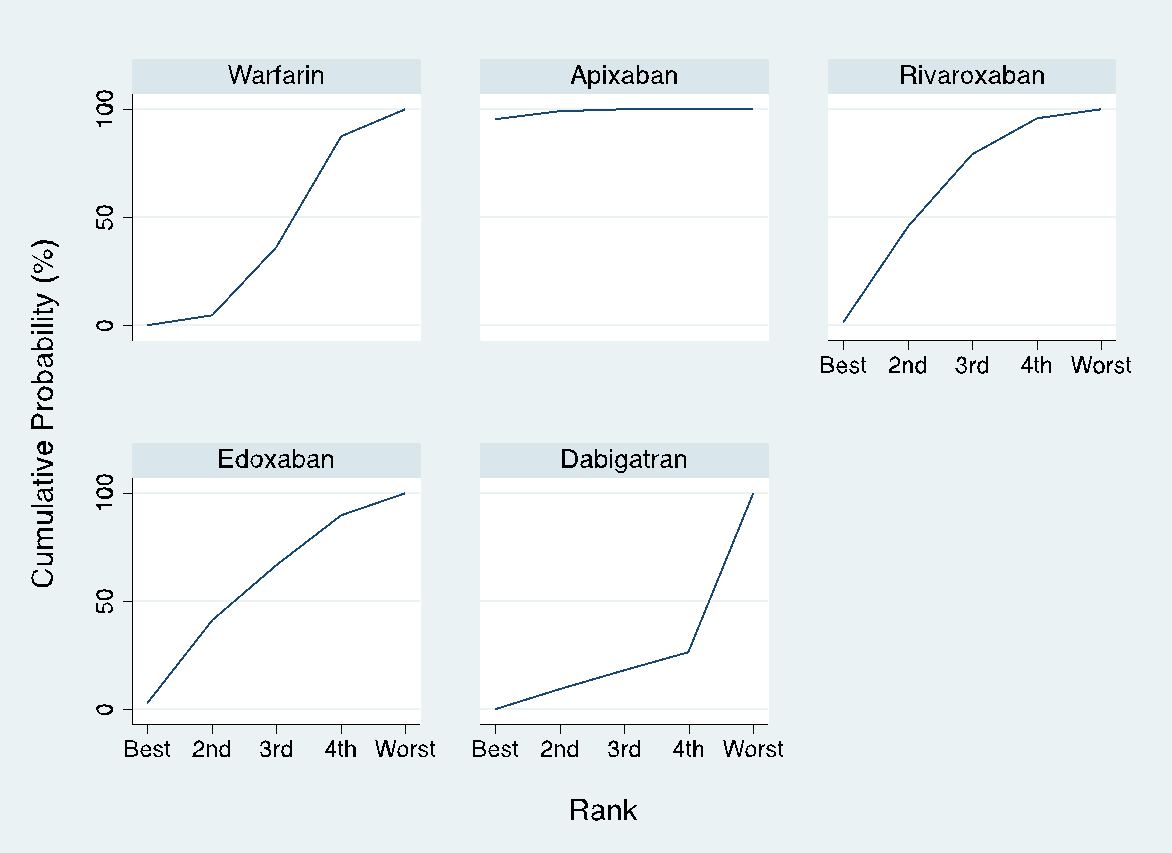** | **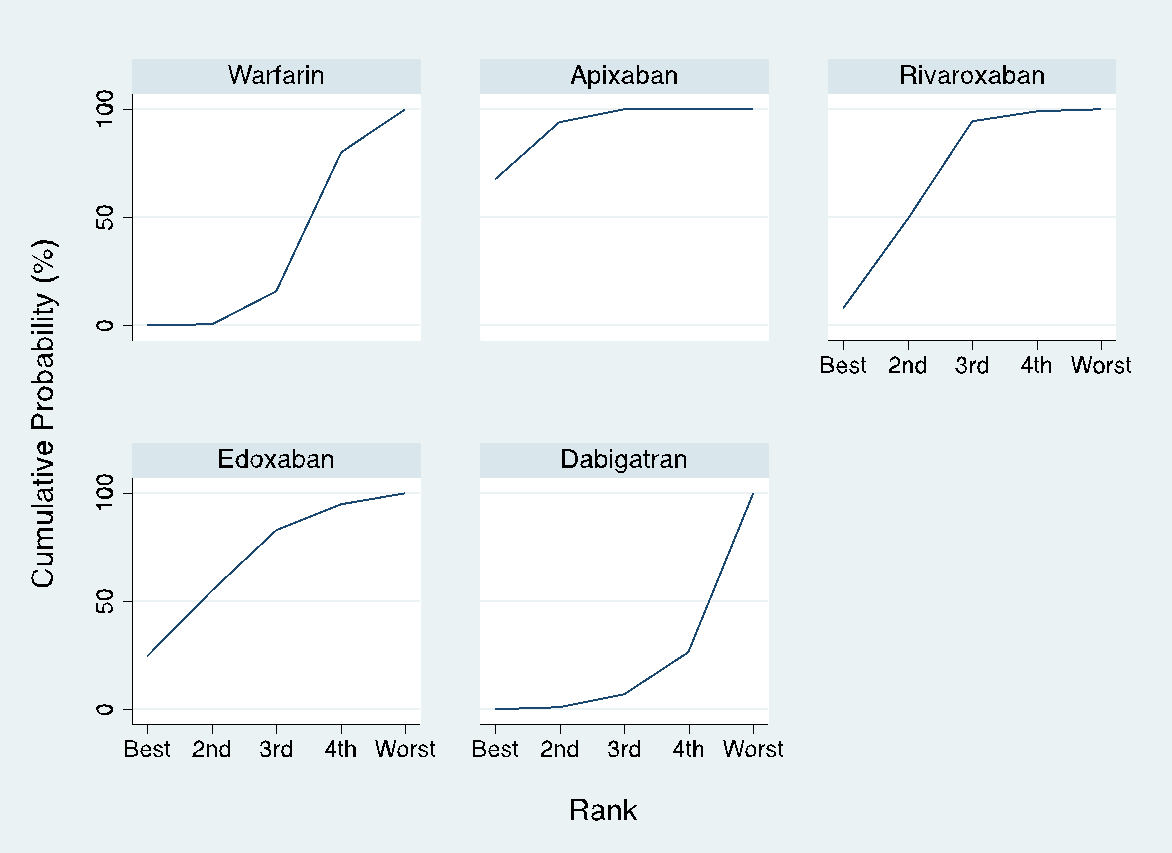** |
| --- | --- | --- |
| All Fracture | Spinal Fracture | Hip fracture |

**Supplementary Figure 2B** Cumulative rank of five anticoagulants for risk of fracture

| **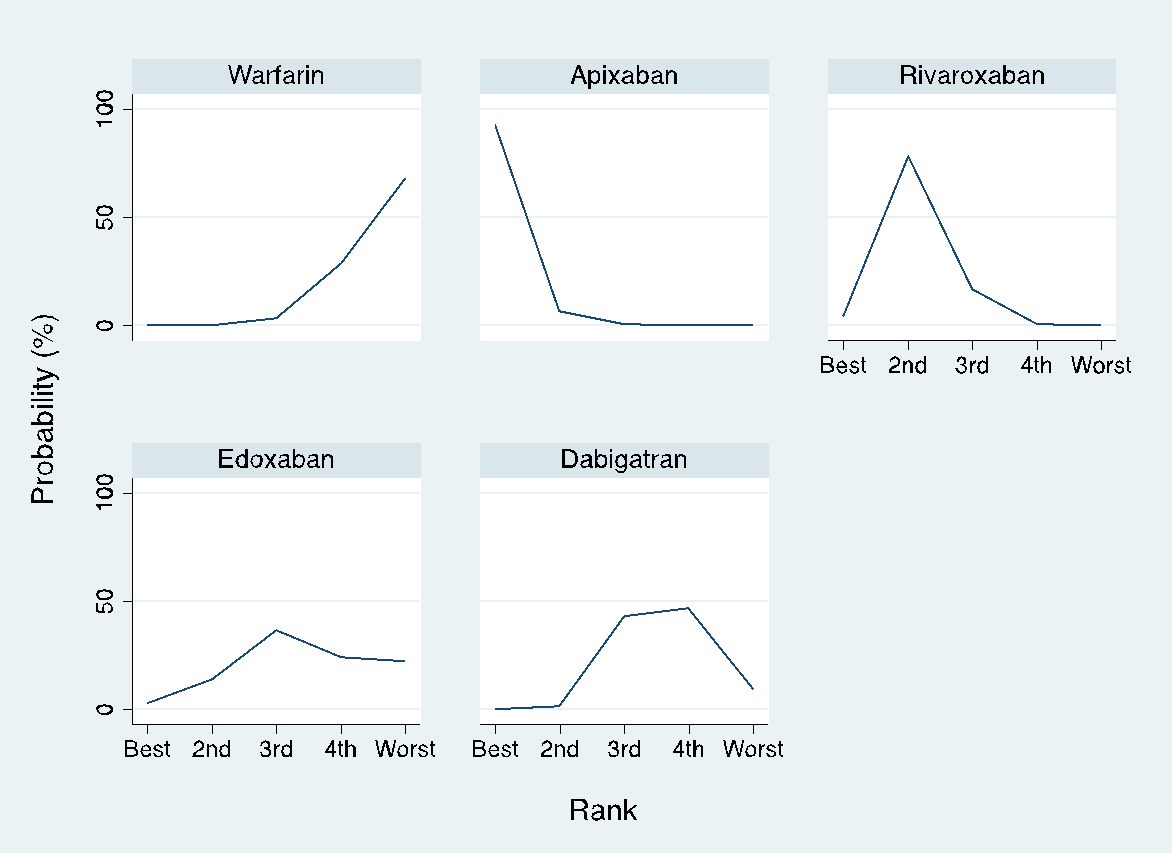** | **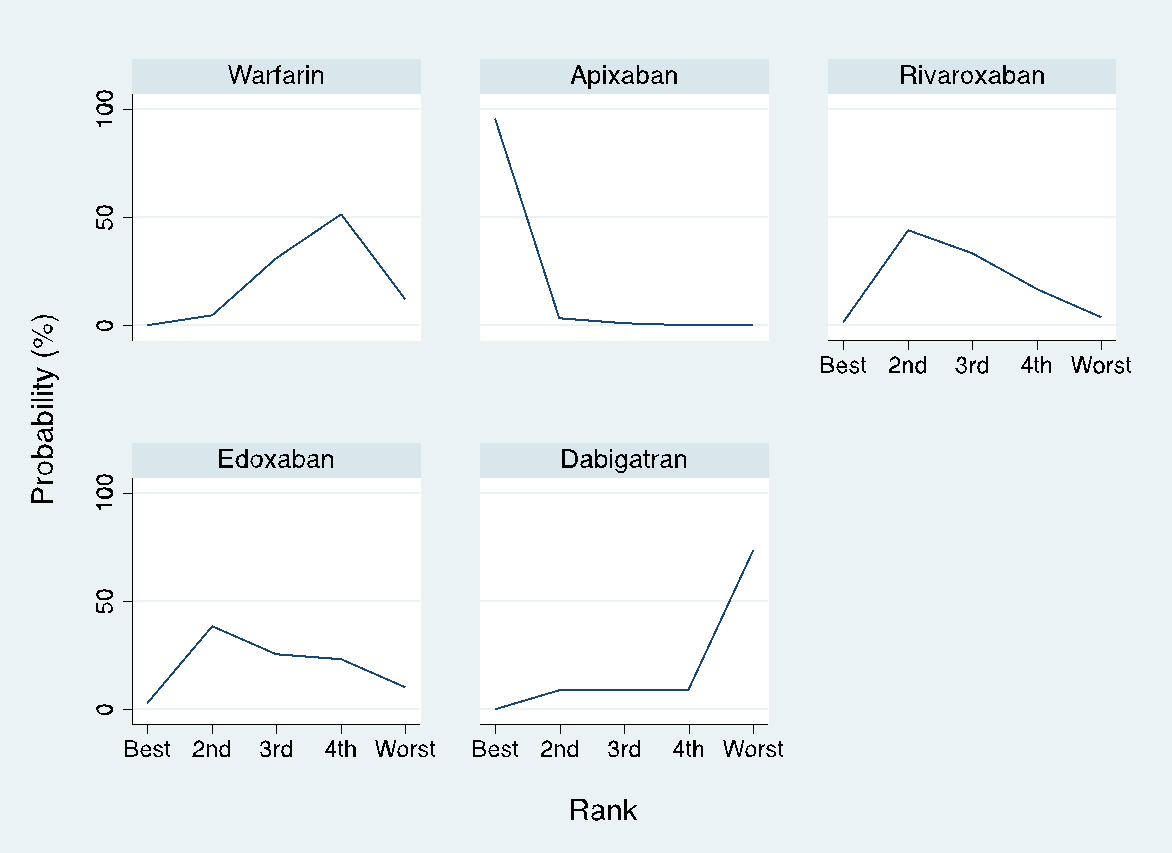** | **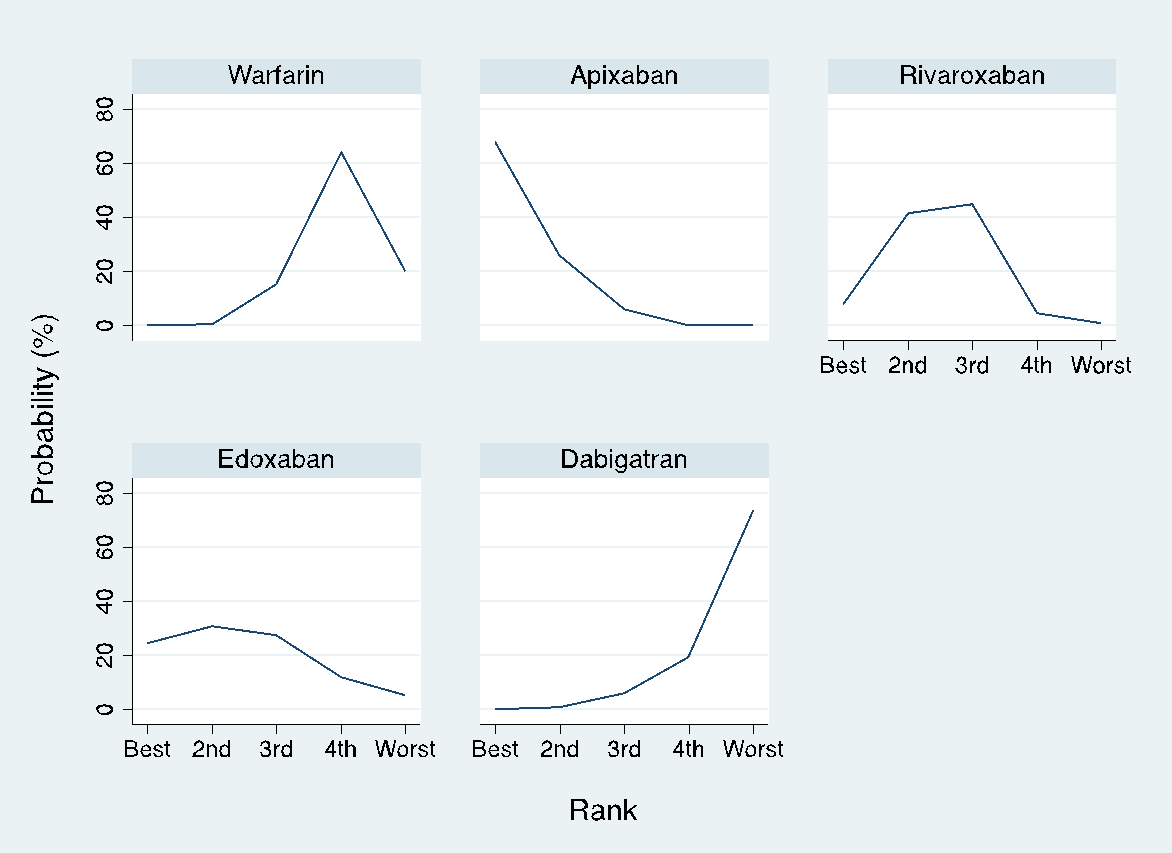** |
| --- | --- | --- |
| All Fracture | Spinal Fracture | Hip fracture |

**Supplementary Figure 3** Forest plot of network meta-analysis results

**Supplementary Figure 3A** Forest plot of network meta-analysis results for all fracture risk. ES, effect size; CI, confidence interval; Prl, prediction interval

**Supplementary Figure 3B** Forest plot of network meta-analysis results for spine fracture risk. ES, effect size; CI, confidence interval; Prl, prediction interval

**Supplementary Figure 3C** Forest plot of network meta-analysis results for hip fracture risk. ES, effect size; CI, confidence interval; Prl, prediction interval

**Supplementary Figure 4:** Publication bias

**Supplementary Figure 4A** Comparison-adjusted funnel plot for the network

A: Apixaban, B: Dabigatran, C: Edoxaban, D: Rivaroxaban, E: Warfarin

| **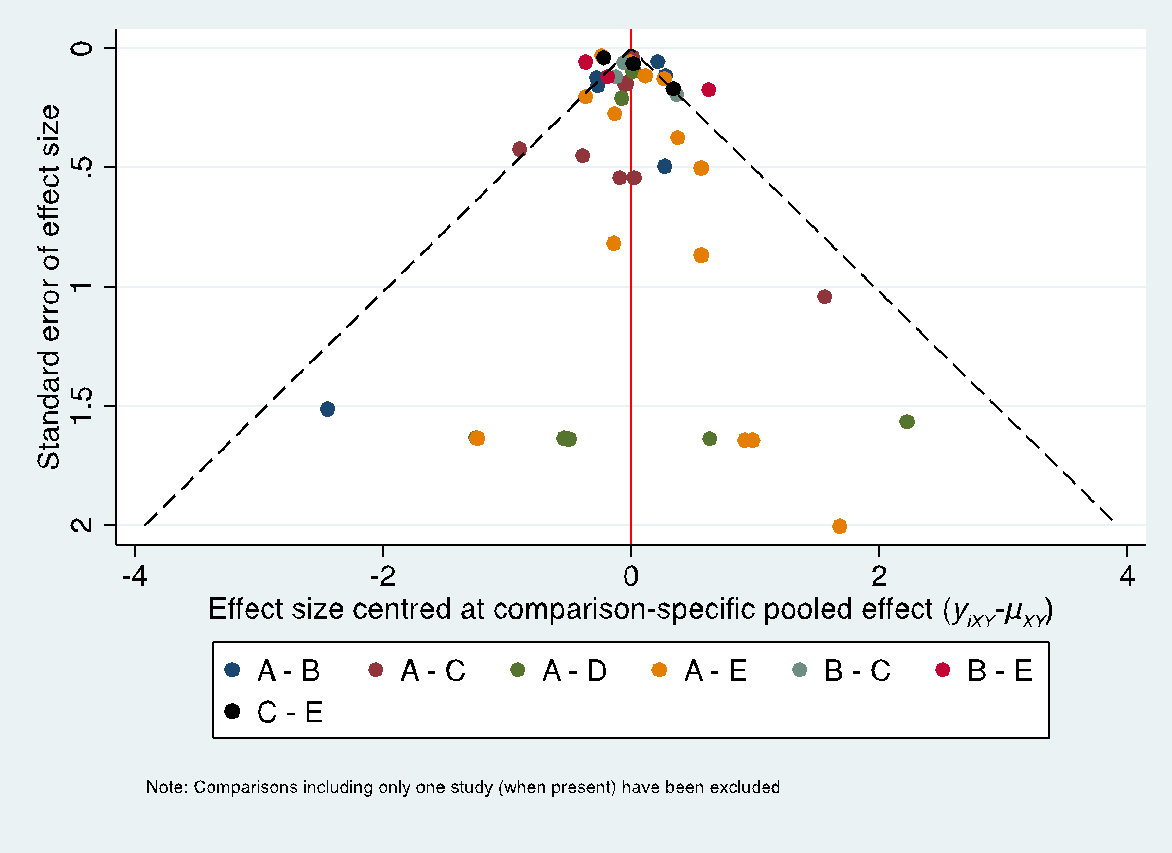** | **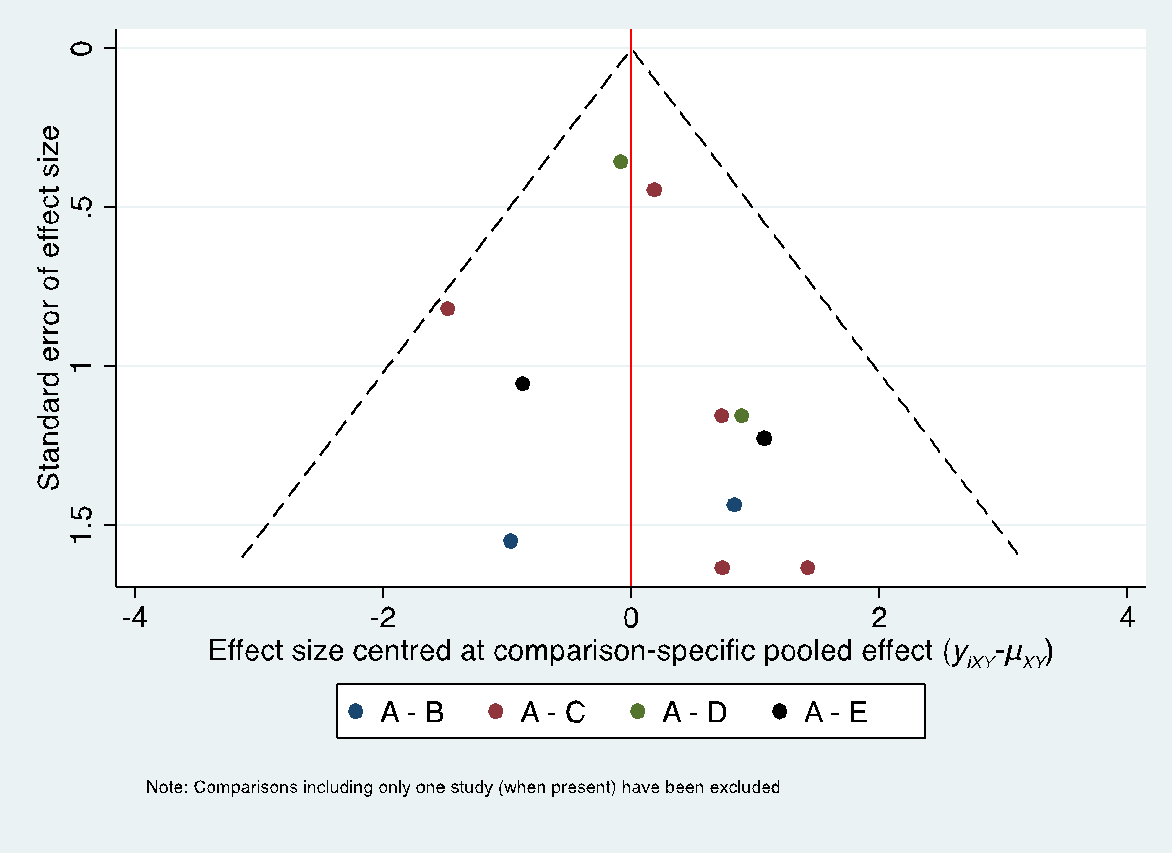** | **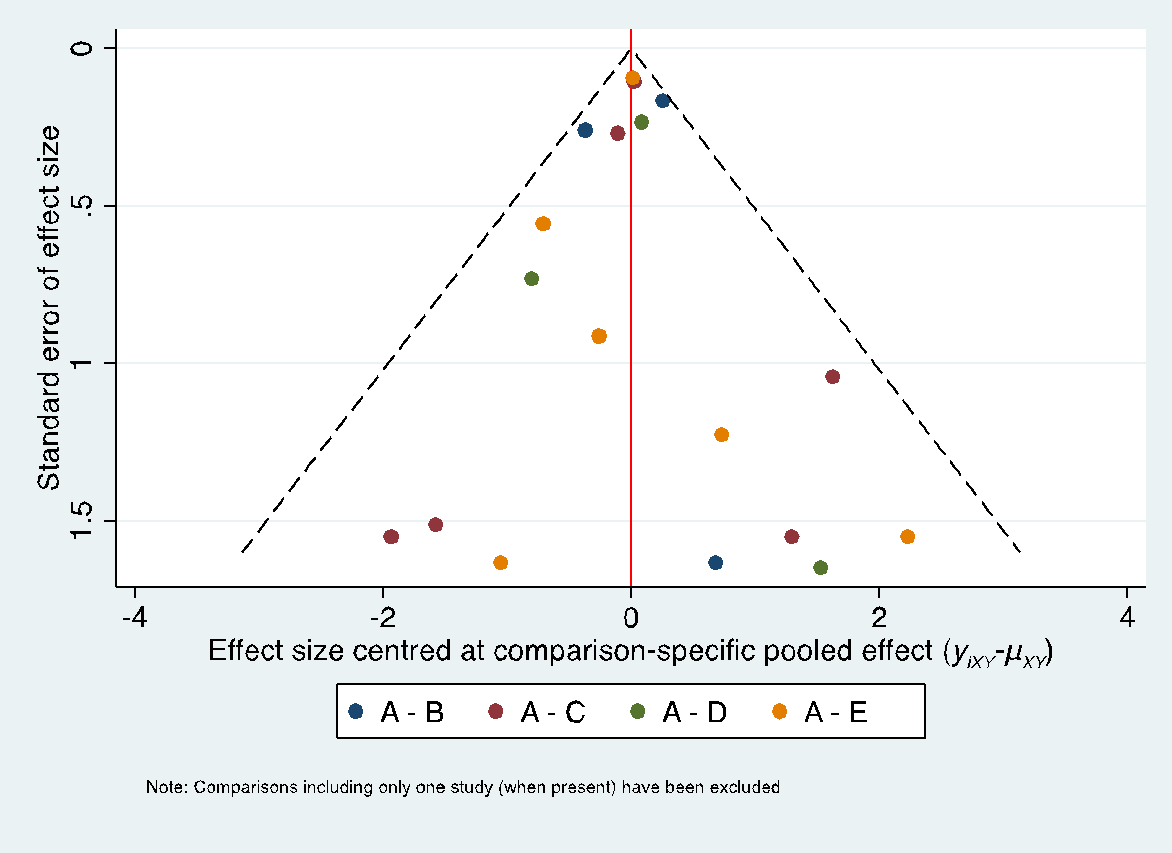** |
| --- | --- | --- |
| All Fracture | Spinal Fracture | Hip fracture |

**Supplementary Figure 4B** Egger’s test for the network

| **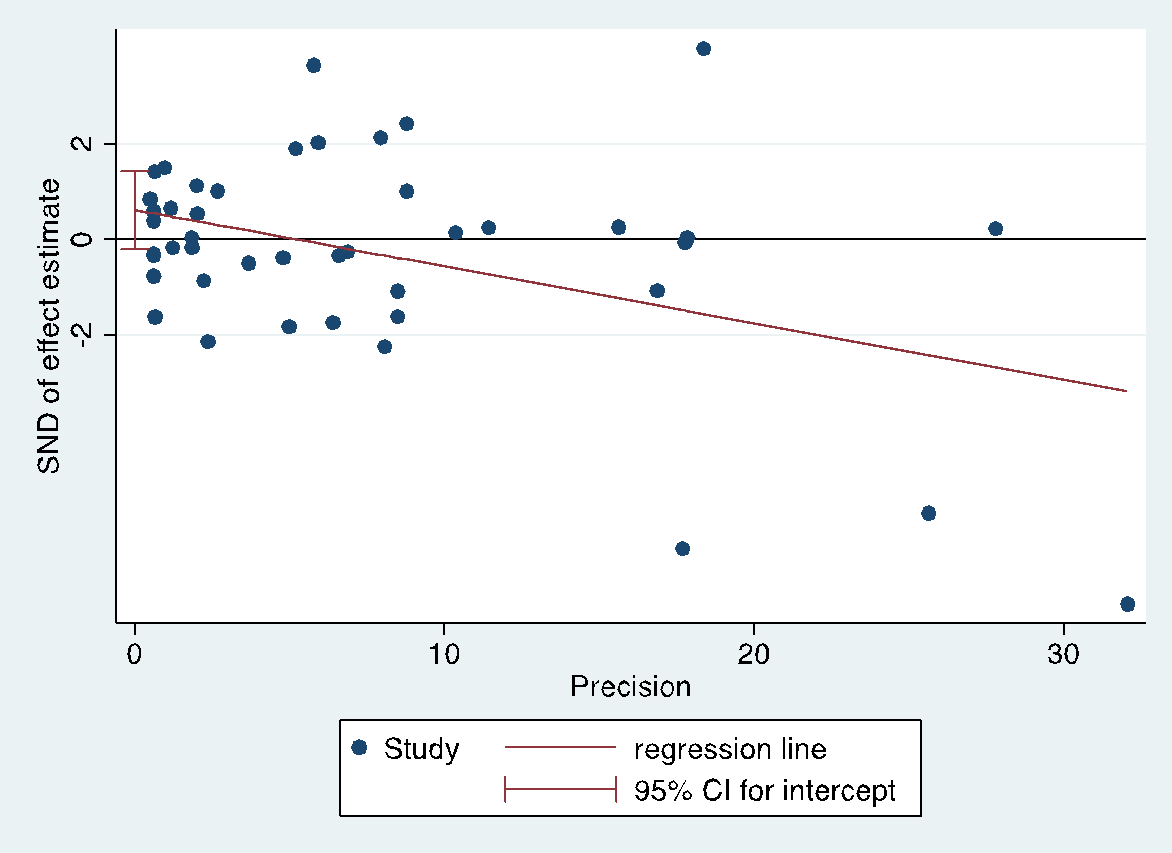** | **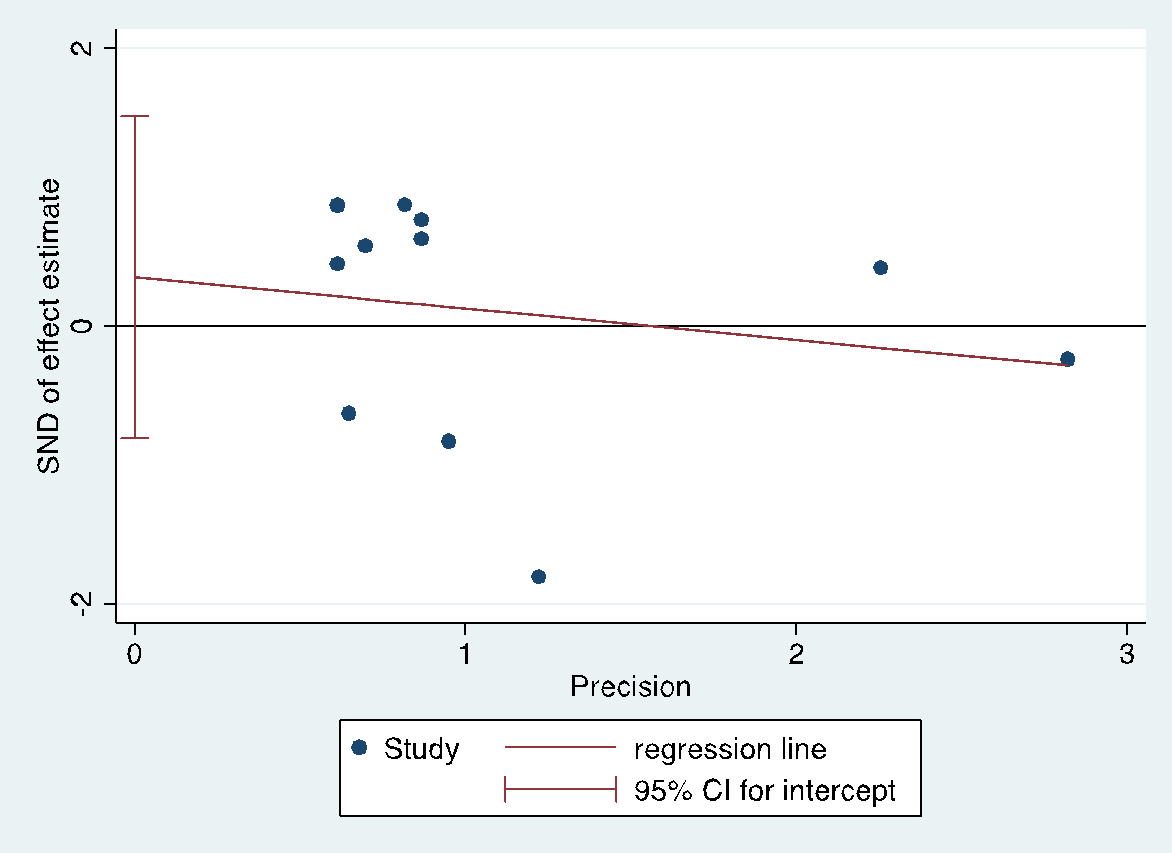** | **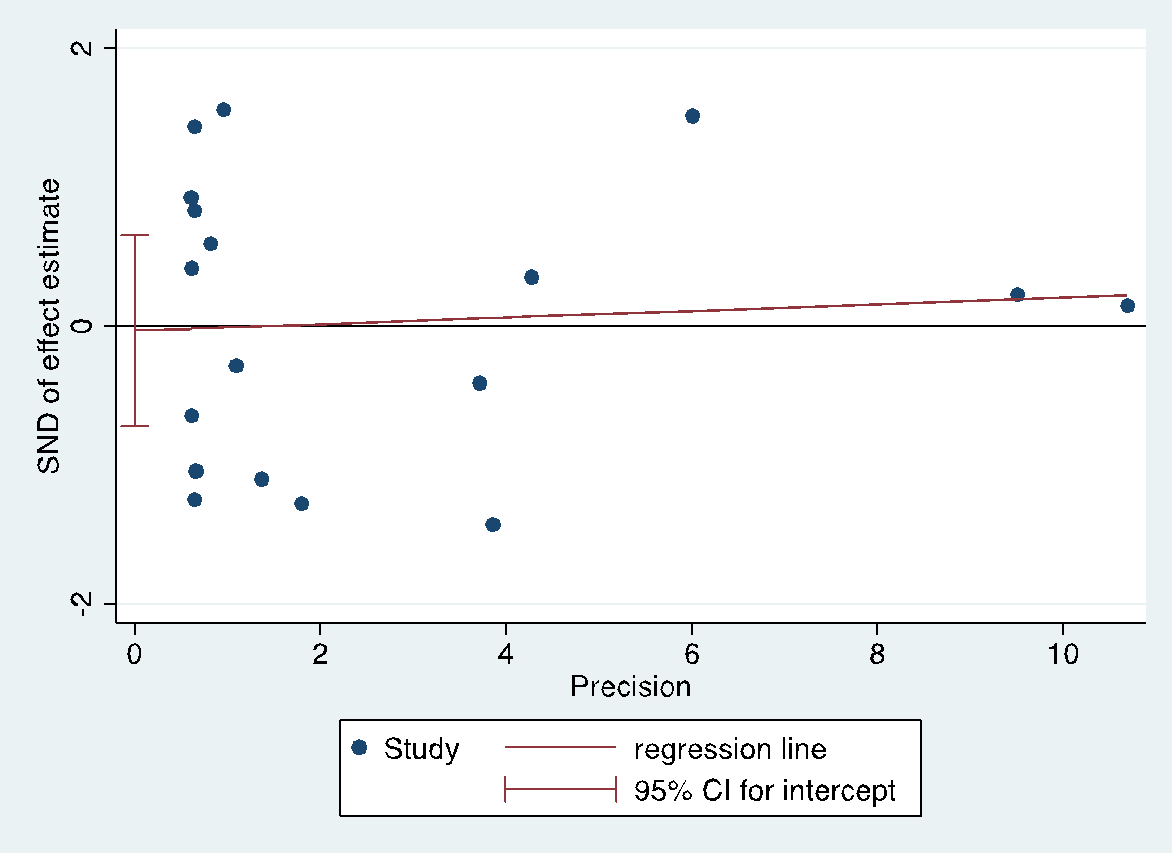** |
| --- | --- | --- |
| All Fracture (P=0.131) | Spinal Fracture (P=0.507) | Hip fracture (P=0.923) |

**Supplementary Table 1** Electronic database search strategy

| **MEDLINE** |
| --- |
| 1. direct oral anticoagulant. AF 2. non-vitamin K antagonist oral anticoagulants. AF 3. novel oral anticoagulants. AF 4. new oral anticoagulants. AF 5. factor Xa inhibitors. AF 6. factor IIa inhibitors. AF 7. Savaysa. AF 8. dabigatran. AF 9. Pradaxa. AF 10. rivaroxaban. AF 11. Xarelto. AF 12. apixaban. AF 13. Eliquis. AF 14. Edoxaban. AF 15. 1 or 2 or 3 or 4 or 5 or 6 or 7 or 8 or 9 or 10 or 11 or 12 or 13 or 14 16. vitamin K antagonist oral anticoagulants. AF 17. warfarin. AF 18. placebo. AF 19. 16 or 17 or 18 20. 15 or 19 21. fracture. AF 22. osteoporosis. AF 23. osteoporotic fractures. AF 24. 21 or 22 or 23 25. Observational Study 26. Randomized Controlled Trial 27. 25 or 26 28. 20 and 24 and 27 |
| **EMBASE** |
| 1. direct oral anticoagulant/exp 2. non vitamin K antagonist oral anticoagulants/exp 3. novel oral anticoagulants/exp 4. new oral anticoagulants/exp 5. factor 10a inhibitor/exp 6. factor 2a inhibitors 7. Savaysa 8. dabigatran/exp 9. Pradaxa 10. rivaroxaban/exp 11. Xarelto 12. apixaban/exp 13. Eliquis 14. Edoxaban /exp 15. 1 or 2 or 3 or 4 or 5 or 6 or 7 or 8 or 9 or 10 or 11 or 12 or 13 or 14 16. vitamin K antagonist oral anticoagulants 17. warfarin/exp 18. placebo/exp 19. 16 or 17 or 18 20. 15 or 19 21. Fracture/exp 22. osteoporosis/exp 23. osteoporotic fractures 24. 21 or 22 or 23 25. controlled clinical trial/lim 26. randomized controlled trial/lim 27. 25 or 26 28. 20 and 24 and 27 |
| **Cochrane CENTRAL** |
| 1. direct oral anticoagulant. AF 2. non-vitamin K antagonist oral anticoagulants. AF 3. novel oral anticoagulants. AF 4. new oral anticoagulants. AF 5. factor Xa inhibitors. AF 6. factor IIa inhibitors. AF 7. Savaysa. AF 8. dabigatran. AF 9. Pradaxa. AF 10. rivaroxaban. AF 11. Xarelto. AF 12. apixaban. AF 13. Eliquis. AF 14. Edoxaban. AF 15. 1 or 2 or 3 or 4 or 5 or 6 or 7 or 8 or 9 or 10 or 11 or 12 or 13 or 14 16. vitamin K antagonist oral anticoagulants. AF 17. warfarin. AF 18. placebo. AF 19. 16 or 17 or 18 20. 15 or 19 21. fracture. AF 22. osteoporosis. AF 23. osteoporotic fractures. AF 24. 21 or 22 or 23 25. Observational Study 26. Randomized Controlled Trial 27. 25 or 26 28. 20 and 24 and 27 |
| **Web of Science** |
| (TS= (Savaysa OR Edoxaban OR Eliquis OR Apixaban OR Xarelto OR Rivaroxaban OR Pradaxa OR Dabigatran) OR TS=(factor IIa inhibitors OR novel oral anticoagulants OR new oral anticoagulants OR Non-vitamin K Antagonist) OR TS=(factor Xa inhibitors) ) AND TS=(fracture) |
| **Scopus** |
| (dabigatran OR pradaxa OR rivaroxaban OR xarelto OR apixaban OR eliquis OR edoxaban OR savaysa OR non-vitamin AND k AND antagonist OR novel AND oral AND anticoagulants OR new AND oral AND anticoagulants OR factor AND xa AND inhibitors OR factor AND iia AND inhibitors ) AND (fracture) |

**Supplementary Table 2** Excluded studies with reasons

| Author | Study Name | Reasons for exclusion |
| --- | --- | --- |
| Monaco 2017 | Safety profile of the direct oral anticoagulants: an analysis of the WHO database of adverse drug reactions.  British Journal of Clinical Pharmacology 2017 | Wrong study design |
| Rao 2018 | Clinical Outcomes and History of Fall in Patients with Atrial Fibrillation Treated with Oral Anticoagulation: Insights From the ARISTOTLE Trial. American Journal of Medicine 2018 | Wrong outcome |
| Gosch 2020 | Outcome of older hip fracture patients on anticoagulation: a comparison of vitamin K-antagonists and Factor Xa inhibitors. Archives of Orthopaedic and Trauma Surgery 2020 | Wrong patient population |
| Coleman 2018 | Effectiveness and safety of rivaroxaban versus warfarin in patients with provoked venous thromboembolism. Journal of Thrombosis and Thrombolysis 2018 | Wrong patient population |
| DeCaterina 2020 | Vitamin K antagonists and osteoporotic fractures: Insights from comparisons with the NOACs, European Heart Journal 2020 | Wrong study design |
| Sugiyama 2017 | Osteoporotic fractures associated with dabigatran vs warfarin. JAMA - Journal of the American Medical Association 2017 | Wrong study design |
| Rodríguez-OllerosRodríguez 2019 | Vitamin K and Bone Health: A Review on the Effects of Vitamin K Deficiency and Supplementation and the Effect of Non-Vitamin K Antagonist Oral Anticoagulants on Different Bone Parameters. Journal of Osteoporosis 2019 | Wrong study design |
| Sugiyama 2017 | Edoxaban Versus Warfarin: Bone Fractures Due to Falling. Journal of the American College of Cardiology 2017 | Wrong study design |
| Lau 2017 | Osteoporotic Fractures Associated With Dabigatran vs Warfarin Reply. JAMA - Journal of the American Medical Association 2017 | Wrong study design |
| Uzoigwe 2020 | Oral Anticoagulants, Proton Pump Inhibitors, and Fracture. Jama Internal Medicine 2020 | Wrong study design |
| Dadwal 2020 | Effect of antithrombotic drugs on bone health. Zeitschrift Fur Gerontologie Und Geriatrie 2020 | Wrong study design |

**Supplementary Table 3** Descriptions of the included studies

| **Study**  **(Author, year)** | **Types of fractures** | **Other comorbidities** | **Other Co-medications** | **Other outcomes** |
| --- | --- | --- | --- | --- |
| Ezekowitz, 2007 | Radius fracture | NA | AAM, HTN medications, CAD medications | Bleeding events, systemic embolic event, abnormal liver function test |
| Connolly, 2009 | Ankle fracture, cervical vertebral fracture, clavicle fracture, compression fracture, facial bones fracture, femoral neck fracture, facture, femur fracture, fibula fracture, foot fracture, forearm fracture, hand fracture; hip fracture; humerus fracture, lower limb fracture, lumbar vertebral fracture, multiple fractures, pathological fracture, patella fracture, pelvic fracture, radius fracture, rib fracture, scapula fracture, skull fracture, spinal compression fracture, spinal fracture, sternal fracture, thoracic vertebral fracture, tibia fracture, upper limb fracture, wrist fracture | NA | AAM, Antiplatelet therapy, HTN medications, CAD medications, PPI | Bleeding events, death, ICH, stroke, VTE, abnormal liver function test |
| Schulman, 2009 | Femur fracture, hip fracture, lower limb fracture, radius fracture, rib fracture, tibia fracture | NA | LMWH | Bleeding events, CAD events, VTE, death, DVT, PE |
| NCT01136408, 2010 | Femur fracture | NA | NA | abnormal liver function test, alkaline phosphatase, bleeding events, adverse events |
| Weitz, 2010 | Clavicle fracture, upper limb fracture | Hypertension | Antiplatelet therapy | Adverse cardiac events, bleeding events, CAD events, death, VTE, stroke |
| EINSTEIN Investigators,2010 | Ankle fracture, clavicle fracture, femoral neck fracture, femur fracture, humerus fracture, radius fracture, rib fracture, spinal compression fracture, thoracic vertebral fracture, ulna fracture, pathological fracture | NA | NA | Bleeding events, death, VTE |
| Chung, 2011 | NA | NA, liver function | Antiplatelet therapy | Bleeding events |
| Granger, 2011 | Acetabulum fracture, ankle fracture, cervical vertebral fracture, clavicle fracture, facial bones fracture, femoral neck fracture, fracture, femur fracture, fibula fracture, foot fracture, forearm fracture, fracture, hand fracture, hip fracture, lower limb fracture, lumbar vertebral fracture, open fracture, patella fracture, pelvic fracture, pubis Fracture, radius fracture, rib fracture, scapula fracture, skull fracture, spinal compression, spinal fracture, sternal fracture, thoracic vertebral fracture, tibia fracture; Ulna fracture, upper limb fracture, wrist fracture, pathological fracture | NA | AAM, HTN medications, CAD medications, NSAIDs. PPI, statin | Death, bleeding events, stroke |
| Patel, 2011 | Ankle fracture, avulsion fracture, cervical vertebral fracture, clavicle fracture, compression fracture, facial bones fracture, femoral neck fracture, femur fracture, fibula fracture, foot fracture, fracture, hand fracture, hip fracture, humerus fracture, lower limb fracture, lumbar vertebral fracture, multiple fractures, pathological fracture, patella fracture, pelvic fracture, pubis fracture, radius fracture, rib fracture, spinal compression fracture, spinal fracture, thoracic vertebral fracture, tibia fracture, upper limb fracture, wrist fracture | PAD, CPD | Antiplatelet therapy | Bleeding events, ICH |
| EINSTEIN-PE  Investigators, 2012 | Ankle fracture, cervical vertebral fracture, facial bones fracture, femoral neck fracture, femur fracture, fibula fracture, foot fracture, hip fracture, humerus fracture, lumbar vertebral fracture, rib fracture, spinal compression fracture, sternal fracture, thoracic vertebral fracture, tibia fracture, upper limb fracture | DVT | NA | Bleeding events, death, malignancy, MI, PE, VTE |
| Hori, 2012 | Femur fracture, fibula fracture, patella fracture, radius fracture, rib fracture, skull fracture, spinal compression fracture, tibia fracture, ulna fracture | NA | NA | Back pain, bleeding events, CHF, diarrhea, death, nasopharyngitis, upper respiratory tract inflammation |
| Hokusai-VTE Investigators, 2013 | Acetabulum fracture, ankle fracture, clavicle fracture, compression fracture, facial bones fracture, femoral neck fracture, femur fracture, fibula fracture, foot fracture, forearm fracture, fractured sacrum, hand fracture, hip fracture, humerus fracture, jaw fracture, lower limb fracture, lumbar vertebral fracture, multiple fractures, pelvic fracture, pubis fracture, radius fracture, rib fracture, spinal compression fracture, spinal fracture, thoracic vertebral fracture, tibia fracture, upper limb fracture, wrist fracture | NA | Heparin | VTE, PE, bleeding events |
| Agnelli, 2013 | Ankle fracture, cervical vertebral fracture, facial bones fracture, femur fracture, hip fracture, humerus fracture, lower limb fracture, lumbar vertebral fracture, pelvic fracture, radius fracture, spinal compression fracture, upper limb fracture, wrist fracture | NA | LMWH, heparin | Death, VTE, bleeding events |
| Giugliano, 2013 | Acetabulum fracture, ankle fracture, cervical vertebral fracture, clavicle fracture, compression fracture, facial bones fracture, femoral neck fracture, femur fracture, fibula fracture, foot fracture, forearm fracture, fracture, fractured coccyx, hand fracture, hip fracture, humerus fracture, jaw fracture, lower limb fracture, lumbar vertebral fracture, multiple fracture, pathological fracture, patella fracture, pelvic fracture, pubis fracture, radius fracture, rib fracture, scapula fracture, skull fracture, spinal compression fracture, spinal fracture, sternal fracture, thoracic vertebral fracture, tibia fracture, ulna fracture, upper limb fracture, wrist fracture | NA | AAM, Antiplatelet therapy, CAD medications | Death, bleeding events, MI, stroke |
| Schulman, 2013 | Acetabulum fracture, ankle fracture, femoral neck fracture, femur fracture, fibula fracture, foot fracture, hand fracture, hip fracture, humerus fracture, lower limb fracture, radius fracture, tibia fracture, upper limb fracture | NA | NA | Bleeding event, CAD, abnormal liver function test, VTE |
| Schulman, 2014 | Femoral neck fracture, hip fracture, humerus fracture, multiple fractures, upper limb fracture | NA | NA | Bleeding event, CAD, abnormal liver function test, MI, PE, VTE or VTE-related death |
| Gibson, 2016 | Compression fracture, femoral neck fracture, hip fracture, humerus fracture, lower limb fracture, pubis fracture, radius fracture, rib fracture, traumatic fracture, upper limb fracture | NA | Antiplatelet therapy | Bleeding event, death, MI, stroke |
| Goette, 2016 | Ankle fracture | Life-threatening bleeding event | NA | Bleeding events, renal function, CAD-related death, ICH, MI, stroke, VTE |
| Piazza, 2016 | Femoral neck fracture, rib fracture | Pulmonary disease, recent bone fracture, renal disease liver disease, | Antiplatelet therapy | Adverse events, bleeding events, ICH |
| Bengtson, 2017 | Hip fracture, pelvic fracture | PAD, CPD, renal/liver disease,  GI bleeding | AAM, Antiplatelet therapy, CAD medications, DM medications, statin | Bleeding events, ICH, MI, stroke |
| Calkins, 2017 | Acetabulum fracture | Left ventricular dysfunction, previous major bleeding, previous GI bleeding or gastritis, renal disease | AAM, NSAIDs, PPI, statins | Bleeding events, VTE, stroke, adverse events |
| Cannon, 2017 | Ankle fracture, clavicle fracture, femoral neck fracture, femur fracture, hand fracture, hip fracture, lumbar vertebral fracture, multiple fracture, pelvic fracture, periprosthetic fracture, pubis fracture, rib fracture, skull fracture, skull fractured base, sternal fracture, upper limb fracture, wrist fracture | NA | NA | Bleeding events, death, ICH, VTE, stroke |
| Lucenteforte, 2017 | Hip fracture, vertebral fracture | NA | NA | NA |
| Norby, 2017 | Hip fracture, pelvic fracture | CPD, dementia, renal/liver, disease,  GI bleeding, PAD | AAM, Antiplatelet therapy, CAD medications, DM medications, statin | Asthma, malignancy, bleeding events, MI, ICH, Stroke |
| Ezekowitz, 2018 | Femur fracture, radius fracture | NA | NA | Bleeding events, stroke, VTE, death |
| Hohnloserm, 2018 | Foot fracture | PAD, valvular heart disease | AAM, Antiplatelet therapy, CAD medications, NSAIDs, PPI, Osteoporosis medications | Death, bleeding events, stroke, VTE |
| Ferro, 2019 | Upper limb fracture | Genetic thrombophilia inflammatory bowel disease, Infection (ear, nose, throat), parenchymal lesion, puerperium, previous VTE, other inflammatory systemic disorder, severe dehydration, surgery | NA | Bleeding event, ICH, VTE |
| Huang, 2020 | Hip fracture, vertebral fracture, humerus/forearm/wrist fracture | Cataract, CKD, CPD, DVT, dementia, depression, epilepsy, hyperthyroidism, hypothyroidism, liver cirrhosis, PAD, RA, Parkinsonism, osteoporosis | Antiepileptics, antiparkinsonian, antipsychotics, antidepressants, antithyroid drugs, anxiolytics, CAD medications, NSAIDs, PPI, statin, thyroxine, Osteoporosis medications | NA |
| Lutsey, 2020 | All fracture, inpatient fracture, hip fracture | Alcohol abuse, CPD, dementia, depression, DVT, GI bleeding, hematological disorder, epilepsy, osteoporosis, PAD, Parkinsonism, renal/liver disease, prior fracture | AAM, Antiplatelet therapy, CAD medications, Osteoporosis medications, DM medications, statin | NA |
| Wang, 2020 | Hip fracture, pelvic fracture | Asthma, dementia, depression, liver disease, alcohol abuse | DM medications | Asthma, malignancy, bleeding events, ICH, MI, stroke |
| Lau, 2020 | Hip fracture, vertebral fracture | CKD, CPD, history of falls,  prior fracture, osteoporosis, liver/renal disease, RA | AAM, HTN medications, antidepressants, Osteoporosis medications | NA |
| **Abbreviations:**  CAD: Cardiovascular disease; CHF: Congestive heart failure; CKD: Chronic kidney disease; CPD: Chronic pulmonary disease; DM: Diabetes mellitus; DVT: Deep vein thrombosis; GI: gastrointestinal; HTN: Hypertension; MI: Myocardial infarction; PAD: Peripheral arterial disease; PE: Pulmonary embolism; RA: Rheumatoid arthritis; VTE: Venous Thromboembolism; AAM: Anti-arrhythmic medications; LMWH: Low molecular weight heparin; PPI: Proton pump inhibitor; DVT: Deep vein thrombosis; ICH: Intracranial hemorrhage; PE: Pulmonary embolism; VTE: Venous Thromboembolism | | | | |

**Supplementary Table 4** Patient characteristics included in the studies

| **Study** | **Treatment** | **CAD**  **(%)** | **Cancer**  **(%)** | **CHF**  **(%)** | **DM**  **(%)** | **HTN**  **(%)** | **Stroke**  **(%)** | **Steroid**  **(%)** | **Smoking**  **(%)** |
| --- | --- | --- | --- | --- | --- | --- | --- | --- | --- |
| Ezekowitz, 2007 | Warfarin | 60 | NA | 34.3 | 21.4 | 70 | 18.6 | NA | 75.7 |
|  | Dabigatran 50 mg | 61 |  | 33.3 | 25.7 | 66.7 | 18 |  | 72.4 |
|  | Dabigatran 150 mg | 63 |  | 31.3 | 27 | 71 | 17.5 |  | 72.3 |
|  | Dabigatran 300 mg | 59.6 |  | 22.4 | 24 | 74 | 16 |  | 72 |
| Connolly, 2009 | Warfarin | 16.1 | NA | 31.9 | 23.4 | 78.9 | 19.8 | NA | NA |
|  | Dabigatran 100 mg | 16.8 |  | 32.2 | 23.4 | 78.8 | 19.9 |  |  |
|  | Dabigatran 150 mg | 16.9 |  | 31.8 | 23.1 | 78.9 | 20.3 |  |  |
| Schulman, 2009 | Warfarin | NA | 4.5 | NA | NA | NA | NA | NA | NA |
|  | Dabigatran 150 mg |  | 5.0 |  |  |  |  |  |  |
| NCT01136408, 2010 | Warfarin | NA | NA | NA | NA | NA | NA | NA | NA |
|  | Dabigatran 110 mg |  |  |  |  |  |  |  |  |
|  | Dabigatran 150 mg |  |  |  |  |  |  |  |  |
| Weitz, 2010 | Warfarin | NA | NA | NA | NA | NA | NA | NA | NA |
|  | Edoxaban 30 mg QD |  |  |  |  |  |  |  |  |
|  | Edoxaban 30 mg BID |  |  |  |  |  |  |  |  |
|  | Edoxaban 60 mg QD |  |  |  |  |  |  |  |  |
|  | Edoxaban 60 mg BID |  |  |  |  |  |  |  |  |
| EINSTEIN Investigators, 2010 | Warfarin + enoxaparin | NA | NA | NA | NA | NA | NA | NA | NA |
|  | Rivaroxaban |  |  |  |  |  |  |  |  |
| Chung, 2011 | Warfarin | NA | NA | 32 | 22.7 | 69.3 | 22.7 | NA | NA |
|  | Edoxaban 30 mg |  |  | 22.8 | 38 | 70.9 | 26.6 |  |  |
|  | Edoxaban 60 mg |  |  | 31.3 | 27.5 | 73.8 | 23.8 |  |  |
| Granger, 2011 | Warfarin | 13.9 | NA | 35.4 | 24.9 | 87.6 | 19.7 | NA | NA |
|  | Apixaban | 14.5 |  | 35.5 | 25.0 | 87.3 | 19.8 |  |  |
| Patel, 2011 | Warfarin  Apixaban | 18.0  16.6 | NA | 62.3  62.6 | 39.5  40.4 | 90.8  90.3 | 54.6  54.9 | NA | NA |
| EINSTEIN-PE Investigators, 2012 | Warfarin | NA | NA | NA | NA | NA | NA | NA | NA |
|  | Rivaroxaban |  |  |  |  |  |  |  |  |
| Hori, 2012 | Warfarin | 8.3 | NA | 40.2 | 37.1 | 79.5 | 63.4 | NA | NA |
|  | Rivaroxaban | 7.0 |  | 41.3 | 39.0 | 79.5 | 63.8 |  |  |
| Hokusai-VTE Investigators, 2013 | Warfarin | NA | NA | NA | NA | NA | NA | NA | NA |
|  | Edoxaban |  |  |  |  |  |  |  |  |
| Agnelli, 2013 | Warfarin+ enoxaparin | NA | 2.8 | NA | NA | NA | NA | NA | NA |
|  | Apixaban |  | 2.5 |  |  |  |  |  |  |
| Giugliano, 2013 | Warfarin | NA | NA | 57.5 | 35.8 | 93.6 | 28.3 | NA | NA |
|  | Edoxaban 30mg |  |  | 56.6 | 36.2 | 93.5 | 28.5 |  |  |
|  | Edoxaban 60mg |  |  | 58.2 | 36.4 | 93.7 | 28.1 |  |  |
| Schulman, 2013 | Warfarin | 6.1 | 4.1 | NA | ^*^7.6 | 36.5 | NA | NA | NA |
|  | Dabigatran | 8.4 | 4.2 |  | ^*^10.5 | 40.7 |  |  |  |
| Schulman, 2014 | Warfarin | NA | 3.9 | NA | NA | NA | NA | NA | NA |
|  | Dabigatran |  | 3.9 |  |  |  |  |  |  |
| Gibson, 2016 | Warfarin | 52.2 | NA | NA | NA | NA | NA | NA | 6.8 |
|  | Rivaroxaban 2.5 mg | 53.2 |  |  |  |  |  |  | 7.9 |
|  | Rivaroxaban 15 mg | 51.5 |  |  |  |  |  |  | 5.2 |
| Goette, 2016 | Warfarin | 18 | NA | 44 | 18 | 78 | 6 | NA | NA |
|  | Edoxaban | 17 |  | 43 | 20 | 78 | 6 |  |  |
| Piazza, 2016 | Warfarin | 14.3 | 10.7 | NA | NA | NA | NA | NA | 17.9 |
|  | Edoxaban | 16.1 | 17.9 |  |  |  |  |  | 21.4 |
| Bengtson, 2017 | Warfarin | 14.7 | 2.3 | 30.4 | 32.1 | 72.9 | 22.3 | NA | NA |
|  | Dabigatran | 13.4 | 1.6 | 24.3 | 28.6 | 75.2 | 20.6 |  |  |
| Calkins, 2017 | Warfarin | 15.1 | NA | 10.7 | 10.7 | 55. | 2.8 | NA | NA |
|  | Dabigatran | 10.1 |  | 9.8 | 9.5 | 52.4 | 3.2 |  |  |
| Cannon, 2017 | Warfarin | 27.3 | NA | NA | 39.9 | NA | 10.2 | NA | NA |
|  | Dabigatran 110 mg | 24.2 |  |  | 36.9 |  | 7.5 |  |  |
|  | Dabigatran 150 mg | 25.4 |  |  | 34.1 |  | 6.8 |  |  |
| Lucenteforte, 2017 | Warfarin | NA | NA | NA | NA | NA | NA | NA | NA |
|  | Direct Xa inhibitor (R,A) |  |  |  |  |  |  |  |  |
|  | Dabigatran |  |  |  |  |  |  |  |  |
| Norby, 2017 | Warfarin | 8.1 | 11.3 | 26.0 | 26.7 | 62.8 | 17.2 | NA | NA |
|  | Rivaroxaban | 7.1 | 10.9 | 23.1 | 25.7 | 66.0 | 15.5 |  |  |
| Ezekowitz, 2018 | Warfarin | NA | NA | NA | 18.7 | 64.4 | NA | NA | NA |
|  | Apixaban |  |  |  | 20.5 | 65.9 |  |  |  |
| Hohnloserm, 2018 | Warfarin | 20.3 | NA | 19.2 | 15.8 | 59.6 | 3.9 | NA | NA |
|  | Edoxaban | 18.6 |  | 17.3 | 13.4 | 60.8 | 5.4 |  |  |
| Ferro, 2019 | Warfarin | NA | 1.7 | NA | NA | NA | NA | NA | NA |
|  | Dabigatran |  | 0 |  |  |  |  |  |  |
| Huang, 2020 | Warfarin  DOAC (D, R, A) | NA | 5.5  5.8 | 28.4  26.3 | 25.9  25.6 | 55.5  56.1 | 25.9  27.4 | 6.0  5.9 | NA |
|  | Warfarin  Dabigatran | 24.2  23.4 | 5.4  5.5 | 21.6  20.2 | 26.5  26.5 | 58.6  58.5 | 31.1  32.7 | 5.4  5.6 | NA |
|  | Warfarin  Rivaroxaban | 26.1  25.4 | 6.1  6.0 | 26.8  25.1 | 25.8  24.6 | 57.3  57.8 | 27.2  27.0 | 6.5  6.1 | NA |
|  | Warfarin  Apixaban | 27.0  26.2 | 6.8  6.5 | 23.5  22.4 | 27.0  27.0 | 59.3  58.8 | 28.9  27.9 | 5.6  5.6 | NA |
| ^#^Lutsey,2020 | Warfarin | 11.2 | 14.0 | 33.0 | 33.2 | 75.5 | 24 | NA | NA |
|  | Dabigatran | 7.2 | 11.1 | 23.8 | 27.7 | 72.5 | 17.7 |  |  |
|  | Rivaroxaban | 8.2 | 12.4 | 24.6 | 28.2 | 75.7 | 17.9 |  |  |
|  | Apixaban | 9.2 | 13.0 | 27.9 | 30.3 | 79.6 | 20.8 |  |  |
| Wang, 2020 | Warfarin | NA | NA | NA | 100 | 36 | NA | NA | 44 |
|  | Rivaroxaban |  |  |  | 100 | 29 |  |  | 43 |
| Lau, 2020 | Warfarin | NA | NA | 30.6 | 30.7 | NA | 27.9 | 9.5 | NA |
|  | Dabigatran |  |  | 19.8 | 29.3 |  | 29.2 | 7.3 |  |
|  | Rivaroxaban |  |  | 19.9 | 27.4 |  | 24.7 | 8.2 |  |
|  | Apixaban |  |  | 23.8 | 28.3 |  | 29.9 | 8.9 |  |
| ^*^Significantly different; ^#^Treatment group before characteristic match  Abbreviations: A, apixaban; D, dabigatran; DOAC, direct oral anticoagulants, E, edoxaban; R, rivaroxaban | | | | | | | | | |

**Supplementary Table 5** Risk of bias assessment according to Newcastle-Ottawa Quality Assessment Scale for cohort studies

| **Study** | **Quality Assessment Criteria** | | | | | | | | |
| --- | --- | --- | --- | --- | --- | --- | --- | --- | --- |
|  | **Selection** | | | | **Comparability** | **Outcome** | | | **Total Score** |
|  | **Representativeness of Exposed Cohort** | **Selection of non Exposed Cohort** | **Ascertainment of Exposure** | **Outcome of Interest** | **Based on Analysis** | **Assessment of Outcome** | **Follow up length** | **Adequacy of follow up of cohorts** |  |
| Lucenteforte 2017 | * | * |  | * | * | * |  |  | 5 |
| Bengtson 2017 | * | * | * | * | * | * | * | * | 8 |
| Norby 2017 | * | * | * | * | * | * | * | * | 8 |
| Huang 2020 | * | * | * | * | * | * | * | * | 8 |
| Lutsey 2020 | * | * | * | * | * | * | * | * | 8 |
| Wang 2020 | * | * | * | * | * | * | * | * | 8 |
| Lau 2020 | * | * | * | * | * | * | * | * | 8 |

- - - 1. **Selection:**
         1. **Representativeness of Exposed Cohort:** Score given if truly representative of the community
         2. **Selection of non Exposed Cohort:** Score given if drawn from the same community
         3. **Ascertainment of Exposure:** Score given if secure record
         4. **Outcome of Interest:** Score given if outcome of interest was not present at start of study
      2. **Comparability:** Score given if study controls for important confounding factors including age and sex
      3. **Outcome:**
         1. **Assessment of Outcome:** Score given if record linkage is correct
         2. **Follow up length:** Score given if follow-up long enough for outcomes to occur
         3. **Adequacy of follow up of cohort:** Score given if complete follow up described in the study

**Supplementary Table 6** Pairwise meta-analysis of risk ratio (95% CI) for fractures

| **Comparison** | | **Pairwise meta-analysis** | **No of participants** | **No. of studies** | **Heterogeneity I^2^** |
| --- | --- | --- | --- | --- | --- |
| All fractures | | | | | |
| Apixaban | Warfarin | 0.60 (0.46 to 0.79)* | 120817 | 6 | 79.6% |
| Dabigatran |  | 0.87 (0.74 to 1.03) | 223007 | 14 | 66.6% |
| Edoxaban |  | 0.87 (0.73 to 1.03) | 26585 | 7 | 0% |
| Rivaroxaban |  | 0.68 (0.64 to 0.72)* | 220559 | 10 | 0% |
| Rivaroxaban | Apixaban | 1.30 (1.06 to 1.60)* | 64240 | 3 | 61.1% |
| Dabigatran |  | 1.58 (0.98 to 2.56) | 64922 | 3 | 93.4% |
| Dabigatran | Rivaroxaban | 1.32 (1.03 to 1.69)* | 87868 | 3 | 89.2% |
| Spinal fracture | | | | | |
| Apixaban | Warfarin | 0.07 (0.01 to 0.55)* | 23866 | 2 | 0% |
| Dabigatran |  | 1.88 (0.28 to 12.51) | 20806 | 2 | 30.9% |
| Edoxaban |  | 0.81 (0.42 to 1.58) | 22311 | 2 | 0% |
| Rivaroxaban |  | 0.69 (0.29 to 1.63) | 25947 | 5 | 16.6% |
| Hip fracture | | | | | |
| Apixaban | Warfarin | 0.60 (0.36 to 1.02) | 95337 | 3 | 52.6% |
| Dabigatran |  | 1.04 (0.87 to 1.25) | 116069 | 6 | 0% |
| Edoxaban |  | 0.74 (0.43 to 1.28) | 22395 | 3 | 8.1% |
| Rivaroxaban |  | 0.71 (0.59 to 1.02) | 113519 | 6 | 16.3% |
| Rivaroxaban | Apixaban | 1.51 (1.06 to 2.14)* | 48085 | 1 | NA |
| Dabigatran |  | 2.20 (1.57 to 3.09)* | 47257 | 1 | NA |
| Dabigatran | Rivaroxaban | 1.46 (1.17 to 1.83)* | 64052 | 1 | NA |

* Statistical significance

**Supplementary Table 7** Mean rank and SUCRA (surface under the cumulative ranking curve) of five anticoagulants for risk of fractures

|  | All fractures | | | Spine fracture | | | Hip fracture | | |
| --- | --- | --- | --- | --- | --- | --- | --- | --- | --- |
| Treatment | SUCRA | PrBest | MeanRank | SUCRA | PrBest | MeanRank | SUCRA | PrBest | MeanRank |
| Apixaban | 98.0 | 92.6 | 1.1 | 98.5 | 95.4 | 1.1 | 90.4 | 67.8 | 1.4 |
| Rivaroxaban | 71.6 | 4.5 | 2.1 | 55.6 | 1.4 | 2.8 | 62.7 | 7.8 | 2.5 |
| Edoxaban | 37.6 | 2.9 | 3.5 | 50.3 | 2.9 | 3.0 | 64.2 | 24.4 | 2.4 |
| Dabigatran | 34.1 | 0 | 3.6 | 13.5 | 0.3 | 4.5 | 8.5 | 0 | 4.7 |
| Warfarin | 8.8 | 0 | 4.6 | 32.1 | 0 | 3.7 | 24.1 | 0 | 4.0 |

PrBest, Probability of best; SUCRA, surface under the cumulative ranking curve.

**Supplementary Table 8** Studies involved in sensitivity analysis

| **Study**  **(Author, year)** | **RCT** | **Given drug dose** | **Af** | **VTE** | **Age> 65** | **Male> 50%** | **Pt No.>5000** |
| --- | --- | --- | --- | --- | --- | --- | --- |
| Ezekowitz, 2007 | * | * | * |  | * | * |  |
| Connolly, 2009 | * | * | * |  | * | * | * |
| Schulman, 2009 | * | * |  | * |  | * |  |
| NCT01136408, 2010 | * | * | * |  | * | * |  |
| Weitz, 2010 | * | * | * |  | * | * |  |
| EINSTEIN Investigators,2010 | * | * |  | * |  | * |  |
| Chung, 2011 | * | * | * |  |  | * |  |
| Granger, 2011 | * | * | * |  | * | * | * |
| Patel, 2011 | * | * | * |  | * | * | * |
| EINSTEIN-PE  Investigators, 2012 | * | * |  | * |  | * |  |
| Hori, 2012 | * | * | * |  | * | * |  |
| Hokusai-VTE Investigators, 2013 | * | * |  | * |  | * | * |
| Agnelli, 2013 | * | * |  | * |  | * | * |
| Giugliano, 2013 | * | * | * |  | * | * | * |
| Schulman, 2013 | * | * |  | * |  | * |  |
| Schulman, 2014 | * | * |  | * |  | * |  |
| Gibson, 2016 | * | * | * |  | * | * |  |
| Goette, 2016 | * | * | * |  |  | * |  |
| Piazza, 2016 | * | * |  | * |  | * |  |
| Bengtson, 2017 |  |  | * |  | * | * | * |
| Calkins, 2017 | * | * | * |  |  | * |  |
| Cannon, 2017 | * | * | * |  | * | * |  |
| Lucenteforte, 2017 |  |  |  |  |  |  | * |
| Norby, 2017 |  |  | * |  | * | * | * |
| Ezekowitz, 2018 | * | * | * |  |  | * |  |
| Hohnloserm, 2018 | * | * | * |  |  | * |  |
| Ferro, 2019 | * | * | * |  |  |  |  |
| Huang, 2020 |  |  | * |  | * | * | * |
| Lutsey, 2020 |  |  | * |  | * | * | * |
| Wang, 2020 |  |  | * |  | * | * |  |
| Lau, 2020 |  |  | * |  | * |  | * |

Abbreviations: Af, atrial fibrillation; RCT, randomized controlled trial; VTE, venous thromboembolism.

**Supplementary Table 9** Subgroup Pairwise meta-analysis of risk ratio (95% CI) for any fracture

| **Comparison** | **Subgroup** | **Pairwise meta-analysis** | **No. of studies** | **Heterogeneity I^2^** |
| --- | --- | --- | --- | --- |
| Apixaban vs. Warfarin | All | 0.62 (0.48 to 0.79)* | 6 | 78% |
|  | NVAF | 0.63 (0.48 to 0.82)* | 5 | 82% |
|  | VTE | 0.46 (0.18 to 1.22) | 1 | NA |
|  | Age< 65 | 1.23 (0.10 to 15.72) | 2 | 66% |
|  | Age>65 | 0.62 (0.48 to 0.80)* | 4 | 85% |
|  | RCT | 0.76 (0.40 to 1.44) | 3 | 38% |
|  | Cohort | 0.56 (0.44 to 0.71)* | 3 | 77% |
|  | N <5000 | 6.94 (0.36 to 134.21)* | 1 | NA |
|  | N >5000 | 0.61 (0.47 to 0.77)* | 5 | 80% |
| Dabigatran vs. Warfarin | All | 0.88 (0.75 to 1.03)* | 14 | 66.5% |
|  | NVAF | 0.89 (0.74 to 1.06) | 10 | 75% |
|  | VTE | 0.83 (0.54 to 1.28) | 4 | 0% |
|  | Age< 65 | 0.61 (0.30 to 1.25) | 5 | 0% |
|  | Age>65 | 0.88 (0.74 to 1.06) | 9 | 80% |
|  | RCT | 0.94 (0.69 to 1.28) | 9 | 0% |
|  | Cohort | 0.73 (0.67 to 0.93)* | 5 | 87% |
|  | N <5000 | 0.59 (0.36 to 0.97)* | 8 | 0% |
|  | N >5000 | 0.92 (0.75 to 1.03) | 6 | 84% |
| Edoxaban vs. Warfarin | - | 0.87 (0.73 to 1.03) | 7 | 0% |
| Rivaroxaban vs. Warfarin | - | 0.68 (0.64 to 0.72)* | 10 | 0% |
| Rivaroxaban vs. Apixaban | - | 1.30 (1.06 to 1.60)* | 3 | 61.1% |
| Dabigatran vs. Apixaban | - | 1.58 (0.98 to 2.56) | 3 | 93.4% |
| Dabigatran vs. Rivaroxaban | - | 1.32 (1.03 to 1.69)* | 3 | 89.2% |

* Statistical significance

Abbreviations: NVAF, non-vulvular atrial fibrillation; NA, not available; RCT, randomized controlled trial; VTE, venous thromboembolism.

**Supplementary Table 10** Subgroup Network meta-analysis of risk ratio (95% CI) for any fracture

| **Comparison** | **Subgroup** | **No. of studies** | **Network meta-analysis (RR, 95% CI)** |
| --- | --- | --- | --- |
| Apixaban vs. warfarin | All | 31 | 0.59 (0.48 to 0.73)* |
|  | RCT only | 24 | 0.77 (0.52 to 1.13) |
|  | Cohort | 7 | 0.53 (0.41 to 0.68)* |
|  | NVAF | 22 | 0.59 (0.58 to 0.75)* |
|  | VTE | 8 | 0.46 (0.18 to 1.22) |
|  | Age < 65 | 14 | 0.60 (0.24 to 1.51) |
|  | Age ≥ 65 | 16 | 0.59 (0.47 to 0.73)* |
|  | Male predominant | 28 | 0.54 (0.44 to 0.65)* |
|  | N <5000 | 19 | 6.97 (0.36 to 135.21) |
|  | N ≥ 5000 | 12 | 0.58 (0.47 to 0.72)* |
| Rivaroxaban vs. warfarin | All | 31 | 0.72 (0.60 to 0.86)* |
|  | RCT | 24 | 0.83 (0.57 to 1.19) |
|  | Cohort | 7 | 0.54 (0.54 to 0.82)* |
|  | NVAF | 22 | 0.70 (0.58 to 0.85)* |
|  | VTE | 8 | 1.23 (0.64 to 2.36) |
|  | Age < 65 | 14 | 1.22 (0.62 to 2.40) |
|  | Age ≥ 65 | 16 | 0.69 (0.57 to 0.84)* |
|  | Male predominant | 28 | 0.71 (0.60 to 0.83)* |
|  | N < 5000 | 19 | 0.93 (0.59 to 1.47) |
|  | N ≥ 5000 | 12 | 0.70 (0.58 to 0.85)* |
| Edoxaban vs. warfarin | All | 31 | 0.88 (0.62 to 1.23) |
|  | RCT | 24 | 0.88 (0.65 to 1.19) |
|  | Cohort | NA | NA |
|  | NVAF | 22 | 0.88 (0.56 to 1.38) |
|  | VTE | 8 | 0.90 (0.60 to 1.35) |
|  | Age < 65 | 14 | 0.93 (0.63 to 1.38) |
|  | Age ≥ 65 | 16 | 0.84 (0.53 to 1.34) |
|  | Male predominant | 28 | 0.88 (0.65 to 1.17) |
|  | N < 5000 | 19 | 0.75 (0.18 to 3.09) |
|  | N ≥ 5000 | 12 | 0.88 (0.63 to 1.25) |
| Dabigatran vs. warfarin | All | 31 | 0.90 (0.75 to 1.07) |
|  | RCT | 24 | 0.87 (0.56 to 1.35) |
|  | Cohort | 7 | 0.87 (0.71 to 1.07) |
|  | NVAF | 22 | 0.90 (0.74 to 1.10) |
|  | VTE | 8 | 0.58 (0.27 to 1.23) |
|  | Age < 65 | 14 | 0.61 (0.30 to 1.25) |
|  | Age ≥ 65 | 16 | 0.90 (0.74 to 1.10) |
|  | Male predominant | 28 | 0.97 (0.82 to 1.14) |
|  | N < 5000 | 19 | 0.59 (0.35 to 0.97)* |
|  | N ≥ 5000 | 12 | 0.93 (0.78 to 1.12) |

* Statistical significance

Abbreviations: NVAF, non-valvular atrial fibrillation; NA, not available; RCT, randomized controlled trial; VTE, venous thromboembolism.

**Supplementary Table 11** Assessment of inconsistency

**Supplemetary Table 11A** Design inconsistency

|  | Chi-Square | P value for test of global inconsistency |
| --- | --- | --- |
| All fracture | 2.15 | 0.5412 |
| Hip fracture | 4.25 | 0.2361 |

**Supplementary Table 11B** side-splitting methods

Side splitting method separated evidence on a particular comparison into direct and indirect evidence and then assessed their differences. Throughout this study, the statistical significance level was set at 5%.
Comparison A B means A vs.B; A: Warfarin, B: Apixaban, C: Rivaroxaban, D: Edoxaban, E: Dabigatran

| All fracture |  | Spinal fracture |  | Hip fracture |  |
| --- | --- | --- | --- | --- | --- |
| Comparison | P-value | Comparison | P-value | Comparison | P-value |
| AB | 0.853 | NA | NA | AB | 0.920 |
| AC | 0.726 | NA | NA | AC | 0.052 |
| AD | NA | NA | NA | AD | NA |
| AE | 0.248 | NA | NA | AE | 0.257 |
| BC | 0.616 | NA | NA | BC | 0.372 |
| BE | 0.215 | NA | NA | BE | 0.423 |
| CE | 0.566 | NA | NA | CE | 0.781 |

**Supplementary Table 12** Assessment of the quality of included studies by GRADE

| **Outcome** | **Quality Assessment Criteria** | | | | | | | |
| --- | --- | --- | --- | --- | --- | --- | --- | --- |
|  | **No. of Studies** | **No. of Patients** | **Inconsistency** | **Indirectness** | **Imprecision** | **Other consideration (Biases)** | **Relative Effect (95% CI)** | **Confidence to Effect Estimates (GRADE)** |
| Overall fracture risk | 31 | 455,343 | Serious | Not serious | Not serious | Not serious | - | High |
| Apixaban versus warfarin in fracture risks | 6 | 35,359 | Serious | Not serious | Not serious | Not serious | 0.59 (0.48, 0.73) | Moderate |
| Rivaroxaban versus warfarin in fracture risks | 10 | 106,996 | Serious | Not serious | Not serious | Not serious | 0.72 (0.60, 0.86) | Moderate |
| Edoxaban versus warfarin in fracture risks | - | 12,975 | Serious | Not serious | Not serious | Not serious | 0.88 (0.62, 1.23) | Moderate |
| Dabigatran versus warfarin in fracture risks | 14 | 78,810 | Serious | Not serious | Not serious | Not serious | 0.90 (0.75, 1.07) | Moderate |

**Supplementary Table 13.** GRADE approach for rating the quality of treatment effect estimate

**Supplementary Table 13A** All fracture

| Comparison | | Direct evidence |  | Indirect evidence |  | Network meta-analysis |  |
| --- | --- | --- | --- | --- | --- | --- | --- |
|  | | MD (95% CI) | Quality of evidence | MD (95% CI) | Quality of evidence | MD (95% CI) | Quality of evidence |
| Apixaban | Warfarin | 0.60 (0.46 to 0.79) | B† | 0.56 (0.32 to 0.98) | B** | 0.59 (0.48 to 0.73) | B |
| Rivaroxaban | Warfarin | 0.68 (0.64 to 0.72) | B* | 0.79 (0.43 to 1.45) | C†† | 0.72 (0.60 to 0.86) | B |
| Edoxaban | Warfarin | 0.87 (0.73 to 1.03) | B* | **-** | - | 0.88 (0.62 to 1.23) | B |
| Dabigatran | Warfarin | 0.87 (0.74 to 1.03) | C†* | 1.25 (0.69 to 2.27) | B** | 0.90 (0.75 to 1.07) | B |
| Rivaroxaban | Apixaban | 1.30 (1.06 to 1.60) | B‡ | 1.09 (0.67 to 1.79) | B** | 1.22 (0.96 to 1.54) | B |
| Edoxaban | Apixaban | **-** | - | 1.49 (1.00 to 2.22) | C†† | 1.49 (1.00 to 2.22) | C |
| Dabigatran | Apixaban | 1.58 (0.98 to 2.56) | B† | 1.19 (0.75 to 1.89) | C†† | 1.53 (1.20 to 1.94) | B |
| Edoxaban | Rivaroxaban | **-** | **-** | 1.22 (0.83 to 1.80) | C†† | 1.22 (0.83 to 1.80) | C |
| Dabigatran | Rivaroxaban | 1.32 (1.03 to 1.69) | B† | 1.14 (0.76 to 1.69) | B** | 1.25 (1.01 to 1.56) | B |
| Dabigatran | Edoxaban | **-** | **-** | 1.03 (0.70 to 1.51) | C†† | 1.03 (0.70 to 1.51) | C |

* Limitation (risk of bias), † heterogeneity ‡imprecision, ¶ inconsistency in side-splitting and down grading, p<0.05, ** contributing direct evidence of

moderate quality, †† contributing evidence of low or very low quality, ‡‡ no first order indirect loop

Quality of evidence: A: high, B: moderate, C: low, D: very low

**Supplementary Table 13B** Spinal fracture

| Comparison | | Direct evidence |  | Indirect evidence |  | Network meta-analysis |  |
| --- | --- | --- | --- | --- | --- | --- | --- |
|  | | MD (95% CI) | Quality of evidence | MD (95% CI) | Quality of evidence | MD (95% CI) | Quality of evidence |
| Apixaban | Warfarin | 0.07 (0.01 to 0.59) | B‡ | **-** | **-** | 0.07 (0.01 to 0.59) | B |
| Rivaroxaban | Warfarin | 0.69 (0.30 to 1.51) | B* | **-** | **-** | 0.69 (0.30 to 1.51) | B |
| Edoxaban | Warfarin | 0.75 (0.26 to 2.15) | B* | **-** | **-** | 0.75 (0.26 to 2.15) | B |
| Dabigatran | Warfarin | 1.93 (0.36 to 10.25) | B* | **-** | **-** | 1.93 (0.36 to 10.25) | B |
| Rivaroxaban | Apixaban | **-** | **-** | 9.91 (0.99 to 98.93) | C†† | 9.91 (0.99 to 98.93) | C |
| Edoxaban | Apixaban | **-** | **-** | 10.84 (1.00 to 118.05) | C†† | 10.84 (1.00 to 118.05) | C |
| Dabigatran | Apixaban | **-** | **-** | 27.80 (1.84 to 419.35) | C†† | 27.80 (1.84 to 419.35) | C |
| Edoxaban | Rivaroxaban | **-** | **-** | 1.09 (0.29 to 4.19) | C†† | 1.09 (0.29 to 4.19) | C |
| Dabigatran | Rivaroxaban | **-** | **-** | 2.80 (0.43 to 18.20) | C†† | 2.80 (0.43 to 18.20) | C |
| Dabigatran | Edoxaban | **-** | **-** | 2.56 (0.36 to 18.03) | C†† | 2.56 (0.36 to 18.03) | C |

* Limitation (risk of bias), † heterogeneity ‡imprecision, ¶ inconsistency in side-splitting and down grading, p<0.05, ** contributing direct evidence of

moderate quality, †† contributing evidence of low or very low quality, ‡‡ no first order indirect loop

Quality of evidence: A: high, B: moderate, C: low, D: very low

**Supplementary Table 13C** Hip fracture

| Comparison | | Direct evidence |  | Indirect evidence |  | Network meta-analysis |  |
| --- | --- | --- | --- | --- | --- | --- | --- |
|  | | MD (95% CI) | Quality of evidence | MD (95% CI) | Quality of evidence | MD (95% CI) | Quality of evidence |
| Apixaban | Warfarin | 0.60 (0.36 to 1.02) | A | 0.94 (0.02 to 50.00) | C†† | 0.59 (0.40 to 0.87) | B |
| Rivaroxaban | Warfarin | 0.71 (0.59 to 1.02) | B* | 3.43 (0.64 to 18.50) | C†† | 0.77 (0.55 to 1.07) | B |
| Edoxaban | Warfarin | 0.74 (0.43 to 1.28) | B* | **-** | - | 0.74 (0.43 to 1.28) | B |
| Dabigatran | Warfarin | 1.04 (0.87 to 1.25) | C* | 1.89 (0.75 to 4.76) | B** | 1.15 (0.70 to 1.67) | B |
| Rivaroxaban | Apixaban | 1.51 (1.06 to 2.14) | B‡ | 0.92 (0.38 to 2.22) | B** | 1.29 (0.84 to 2.00) | B |
| Edoxaban | Apixaban | - | **-** | 1.26 (0.84 to 2.00) | C†† | 1.26 (0.65 to 2.43) | C |
| Dabigatran | Apixaban | 2.20 (1.57 to 3.09) | A | 1.30 (0.43 to 4.00) | C†† | 1.94 (1.24 to 3.02) | B |
| Edoxaban | Rivaroxaban | - | - | 0.97 (0.51 to 1.83) | C†† | 0.97 (0.51 to 1.83) | C |
| Dabigatran | Rivaroxaban | 1.46 (1.17 to 1.83) | A | 1.72 (0.61 to 4.76) | B** | 1.50 (1.00 to 2.24) | A |
| Dabigatran | Edoxaban | **-** | - | 1.54 (0.81 to 2.94) | C†† | 1.54 (0.81 to 2.94) | C |

* Limitation (risk of bias), † heterogeneity ‡imprecision, ¶ inconsistency in side-splitting and down grading, p<0.05, ** contributing direct evidence of

moderate quality, †† contributing evidence of low or very low quality, ‡‡ no first order indirect loop

Quality of evidence: A: high, B: moderate, C: low, D: very low
